# Binding of the general anesthetic sevoflurane to ion channels

**DOI:** 10.1371/journal.pcbi.1006605

**Published:** 2018-11-26

**Authors:** Letícia Stock, Juliana Hosoume, Leonardo Cirqueira, Werner Treptow

**Affiliations:** Laboratório de Biologia Teórica e Computacional (LBTC), Universidade de Brasília DF, Brasil; Heidelberg Institute for Theoretical Studies (HITS gGmbH), GERMANY

## Abstract

The direct-site hypothesis assumes general anesthetics bind ion channels to impact protein equilibrium and function, inducing anesthesia. Despite advancements in the field, a first principle all-atom demonstration of this structure-function premise is still missing. We focus on the clinically used sevoflurane interaction to anesthetic-sensitive Kv1.2 mammalian channel to resolve if sevoflurane binds protein’s well-characterized open and closed structures in a conformation-dependent manner to shift channel equilibrium. We employ an innovative approach relying on extensive docking calculations and free-energy perturbation of all potential binding sites revealed by the latter, and find sevoflurane binds open and closed structures at multiple sites under complex saturation and concentration effects. Results point to a non-trivial interplay of site and conformation-dependent modes of action involving distinct binding sites that increase channel open-probability at diluted ligand concentrations. Given the challenge in exploring more complex processes potentially impacting channel-anesthetic interaction, the result is revealing as it demonstrates the process of multiple anesthetic binding events alone may account for open-probability shifts recorded in measurements.

## Author summary

General anesthetics are central to modern medicine, yet their microscopic mechanism of action is still unknown. Here, we demonstrate that a clinically used anesthetic, sevoflurane, binds the mammalian voltage-gated potassium channel Kv1.2 effecting a shift in its open probability, even at low concentrations. The results, supported by recent experimental measurements, are promising as they demonstrate that the molecular process of direct binding of anesthetic to ion channels play a relevant role in anesthesia.

## Introduction

Volatile and injected general anesthetics encompass a diverse array of small and uncharged chemotypes including haloalkanes, haloethers and alkylphenols. Despite efforts reaching back over a century, clarification of their microscopic mechanism in general anesthesia has proven difficult and wanting. A favored hypothesis proposes that ion channels in the brain are implicated, among which members of ionotropic neurotransmitter receptors, voltage-gated and non-gated ion channels are best-known players [1–3]. Primary exemplars are the Cys-loop nicotinic acetylcholine and γ-aminobutyric acid class A receptors, the voltage-gated sodium and potassium channels, and the tandem pore potassium channels. An extensive series of electrophysiological studies corroborate the hypothesis by demonstrating a range of effects, from inhibition to potentiation, of general anesthetics on the various receptor targets. Beyond these electrophysiological studies of reductionist systems, the current view has gained additional support from gene knockout experiments demonstrating for some of these channels the *in vivo* role on a clinically-relevant anesthetic outcome. For instance, the knockout of the non-gated tandem pore potassium channel trek-1 produces an animal model (Trek1-/-) resistant to anesthesia by inhalational anesthetics [4].

How general anesthetics modulate ion channels to account for endpoints of anesthesia must at some point build on understanding electrophysiological data in the context of ligand binding, a reasoning that has driven mounting efforts in the field. Currently, though not refuting other molecular processes likely to contribute to anesthetic action [5–7], crystallography and molecular dynamics studies support that anesthetics bind ion channels at clinical concentrations [8–16]. Binding interactions have been evidenced in anesthetic containing systems of mammalian voltage and ligand-gated channels, as well as bacterial channel analogs. Specifically, partitioning of anesthetics in the membrane core allows it to access and bind multiple transmembrane (TM) protein sites, featuring single or multiple occupancy states–a process that might depend further on chemotypes, channel types and conformations. Although some progress has been made in validating one or more aspects of the direct-site hypothesis, a first-principle demonstration that anesthetics bind ion channels to affect protein equilibrium and function as recorded in experiments is still unaccounted for.

Here, we focus our efforts on the haloether sevoflurane and its molecular interaction to Kv1.2, a mammalian voltage-gated potassium channel. Experimental work demonstrates that sevoflurane potentiates the channel in a dose-dependent manner [3,17,18]. Effects on current tracings include a leftward shift in the channel’s conductance-voltage relationship and an increased maximum conductance. As extensively discussed in these past publications, at least two molecular mechanisms are expected to be involved in Kv channels potentiation by sevoflurane. One mechanism (i) might involve sites allosterically coupled to the electromechanical transduction directly responsible for controlling voltage-dependent gating. The other (ii) might involve distinct sites, which could modulate the channel’s pore region and influence the stability of the conductive state and/or the unitary conductance. Here, we are interested in the investigation of mechanism (i) and its underlying structural hypothesis that sevoflurane binds the channel’s open and closed states to impact protein equilibrium and therefore its voltage dependence. Among all other aspects that might impact channel-anesthetic interactions in general, our specific goal is to determine if sevoflurane binds the well-characterized open-conductive (**O**) and resting-closed (**C**) structures of Kv1.2 [19,20] in a conformation-dependent manner to impact its voltage-dependent open probability as recorded experimentally. Very recently, we have put forth an innovative structure-based study [21] dealing with the concentration-dependent binding of small ligands to multiple saturable sites in proteins to show that sevoflurane binds the open-pore structure of Kv1.2 at the S4S5 linker and the S6P-helix interface–a result largely supported by independent photolabeling experiments [22,23]. To our current goal, we aim therefore at extending these calculations to investigate sevoflurane interactions with the entire channel TM-domain and, more importantly, to resolve any conformational dependence in its binding process to channel structures **C** and **O**. In the following sections, we first provide the theoretical framework to study sevoflurane binding to a specific channel conformation under equilibrium conditions. A state-dependent strategy is put forward to describe anesthetic binding in terms of occupancy states of all identified potential channel binding sites, embodying both concentration and multiple sites saturation effects. The strategy is then generalized to account for ligand impact in the **C**-**O** equilibrium, allowing for reconstruction of voltage-dependent open probabilities of the channel at various ligand concentrations. Anticipating our results, we find that sevoflurane binds Kv1.2 structures at multiple sites under saturation and concentration effects. Despite a similar pattern of molecular interactions, binding of sevoflurane is primarily driven towards the open-conductive state shifting leftward the open probability of the channel at diluted ligand concentrations.

## Results

### Binding of anesthetics to multiple channel sites

**Fig 1:**
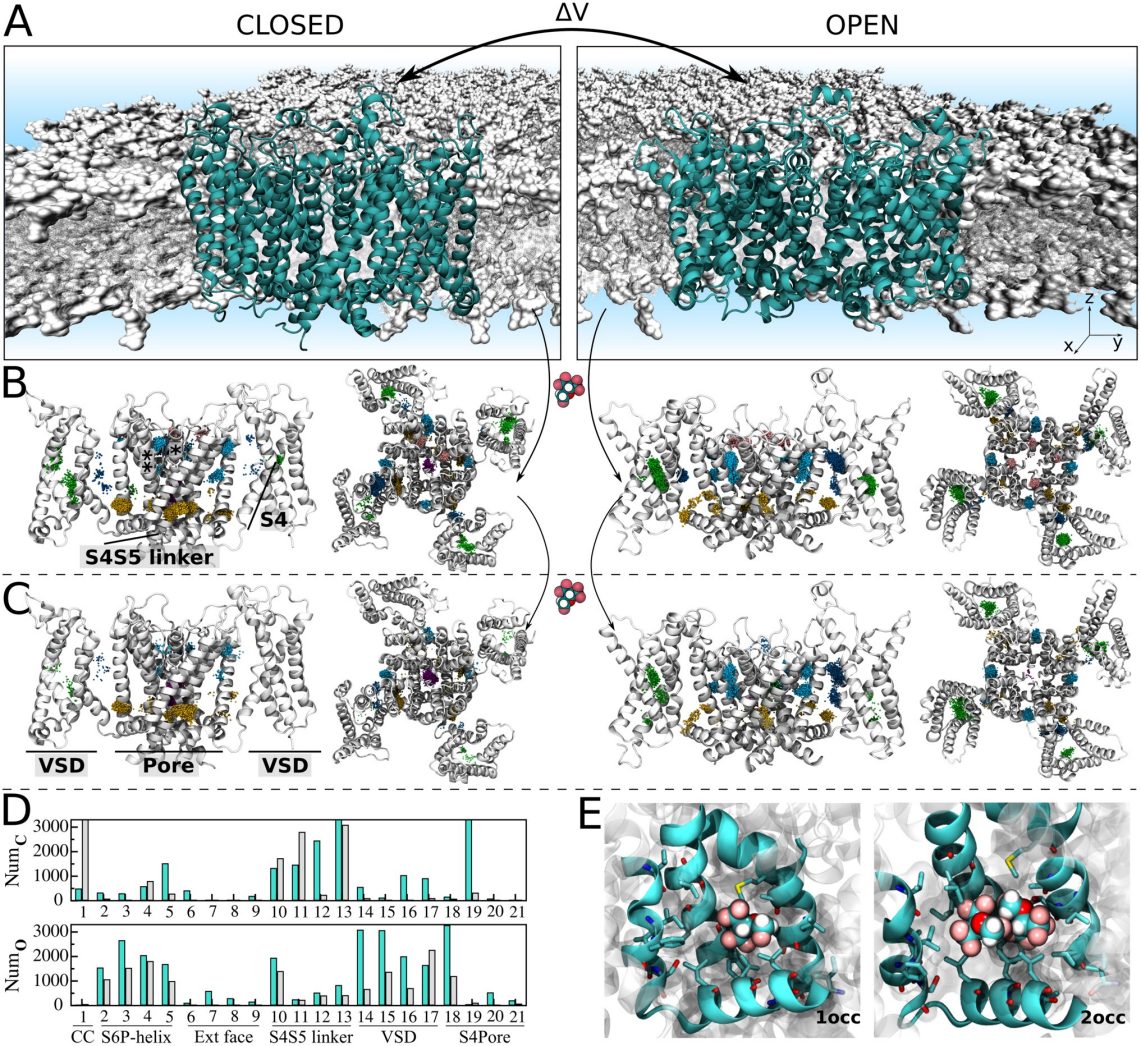
Resolution of sevoflurane sites at the homotetrameric Kv1.2 structures C and O. (A) Atomistic systems containing Kv1.2 structures (cyan) embedded in a fully-hydrated lipid bilayer (gray) were MD simulated to produce molecular ensembles considered for flexible docking calculations. (B) Docking poses for singly-occupied sites. Shown is the ensemble-average channel structures **C** and **O**, along with the set of centroid configurations of sevoflurane (points) determined from docking. Centroid configurations of sevoflurane were clustered as a function of their location on the channel structures, that is within the voltage-sensor (green), at the S4S5 linker (yellow), at the S4Pore (dark blue) and S6P-helix (light blue) interfaces, at the central cavity (violet) and extracellular face (pink). Each of these clusters was treated as an interaction site *j* for sevoflurane with volume *δV*_*j*_. (C) Following another round of docking calculations starting from structures in (B), solutions for doubly occupied sites were resolved by determining if volumes *δV*_*j*_ could accommodate the centroid positions of two docked ligands at once. For better annotation, helices S4, S4S5 linker, S6 (**) and P-helix (*), as well as the pore and VSD domains are indicated in structures in (B) and (C). (D) Per site number of docking solutions for single (cyan) and double (gray) ligand occupancy. (E) Representative molecular structure resolved from docking. Voltage-sensor domains in two opposing channel subunits are not depicted for clarity in (B) and (C) lateral views.

We applied large-scale and flexible docking calculations to solve sevoflurane interactions to Kv1.2 structures ***X*** ≡ {***C***, ***O***} (Fig 1). A total of ~ 6,000 docking solutions was generated per channel conformation and clustered into 21 ligand interaction sites. The interaction sites spread over the transmembrane region of the channel at the S4S5 linker, S6P-helix interface and at the extracellular face, next to the selectivity filter. Further docking sites were resolved within the voltage-sensor, at the S4Pore interface and within the channel central cavity. Re-docking of sevoflurane generated in turn a total of ~ 13,000 solutions per channel conformation, solving the interaction of two ligands for all sites but the extracellular face.

From the docking ensembles, there are up to 2 × 3^21^ channel occupancy states that might contribute to sevoflurane binding and functional effects. To quantitatively evaluate this, we performed an extensive series of decoupling FEP calculations to estimate the per-site binding affinity for one and two bound ligands against the channel structures (*cf*. Computational Methods for details). Here, FEP calculations started from equilibrium ligand-bound channel structures, embedded in an explicit water-membrane environment (*cf*. RMSD analysis in S1 Fig). For the purpose of improving statistics, FEP estimates and the associated statistical errors were determined from at least two independent decoupling runs. Calculations were performed over ~ 7.0 ns per replica, per site, per conformation, to converge FEP estimates; in a total MD simulation time of ~ 2.0 μs. S2 Fig shows the effectively sampled configuration space in FEP calculations for each of the channel structures. Systematic errors related to lack of site rehydration or relipidation during ligand decoupling were ruled out in S3 Fig showing equilibrium-like lipid or water coordination numbers of the channel structure at the final stages of FEP. Under these technical details, per-site equilibrium binding constants were quantified relative to a homogeneous and diluted aqueous solution occupied by ligands, with an excess chemical potential of $\bar{\mu }=0.10\pm 0.09kcal.mol^{-1}$. As shown in S1 and S2 Tables, per-site binding constants are heterogeneous and take place over a diverse range, *i*.*e*. 10^−8^ (mM^-1^) -10^+2^ (mM^-2^). There is however a decreasing trend of affinities involving sites respectively at the S4S5 linker, S4Pore and S6P-helix interfaces, voltage sensor, central cavity and extracellular face.

**Fig 2:**
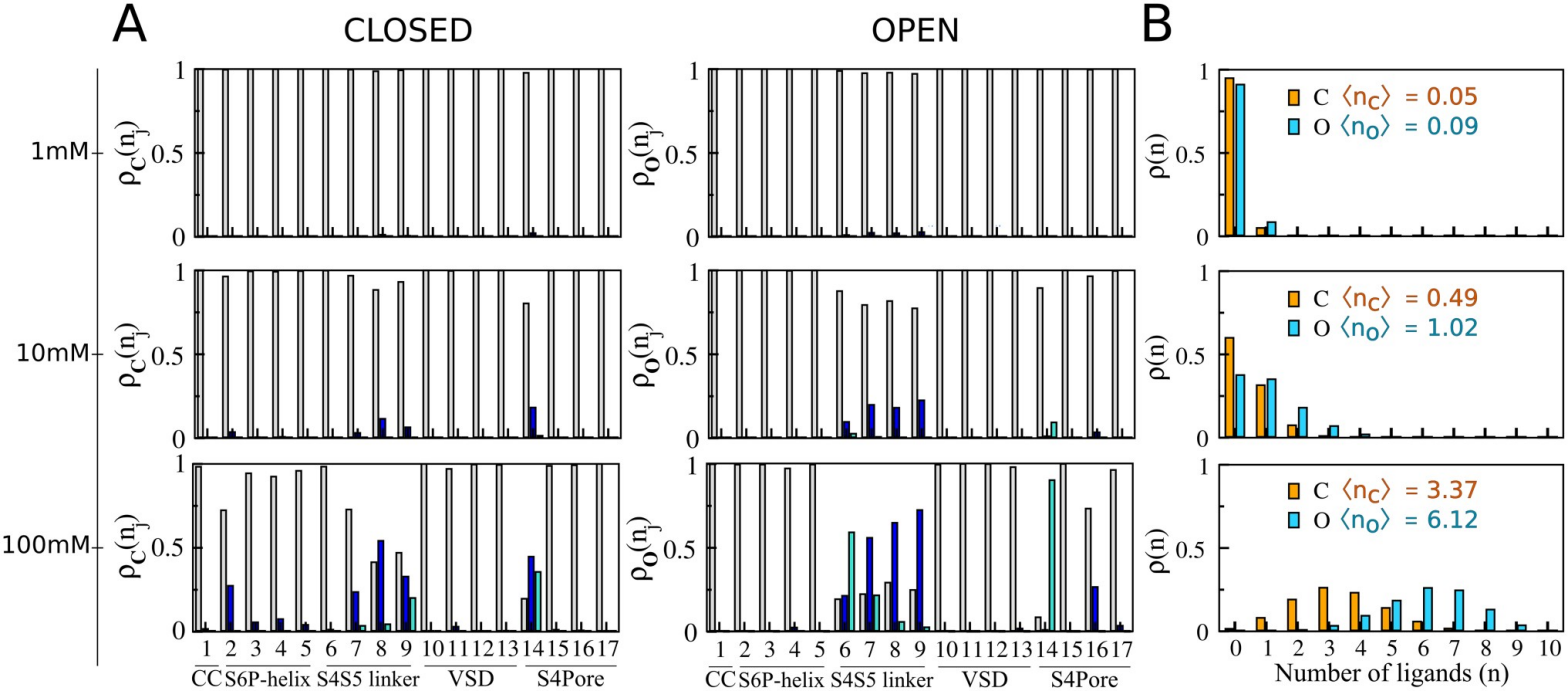
C and O state-dependent binding probabilities for different concentrations of sevoflurane at the reservoir. (A) Marginal probabilities *ρ*_***X***_(*n*_*j*_) of site *j*, for *n*_*j*_ = 0 (gray), *n*_*j*_ = 1 (blue) and *n*_*j*_ = 2(cyan). Marginals at the extracellular face of the channel are negligible for every structure/concentration. (B) Probabilities *ρ*_***X***_(*n*) for macrostates $O_{X}^{*}(n)$ mapping an ensemble of accessible states $O_{X}^{*}(n_{1},\ldots ,n_{s})$ in which *n* ligands bind the receptor regardless their specific distributions over the binding sites. Here, *ρ*_***X***_(*n*_*j*_) and *ρ*_***X***_(*n*) were computed by coarse-graining over state probabilities in S4 Fig (*cf*. Computational Methods for details). Average number ⟨*n*_***X***_⟩ of bound ligands as a function of the reservoir concentration is indicated in (B).

To determine if sevoflurane binds channel structures ***X*** ≡ {***C***, ***O***} at clinically relevant concentrations, we computed binding probabilities *ρ*_***X***_(*n*_1_,…,*n*_*s*_) for dilute concentrations of the ligand in solution, *i*.*e*. 1mM, 10mM and 100mM. Equilibrium constants *K*_***X***_(*n*_1_,…,*n*_*s*_) for every occupancy state of the channel were then reconstructed from the per-site affinities to determine state probabilities via eq (2). Here, estimates of *K*_***X***_(*n*_1_,…,*n*_*s*_) were determined for the condition of independent binding sites, as the minimum site-to-site distances of ~15 Å demonstrated their non-overlap distributions in each of the channel structures (*cf*. Computational Methods for details). At low 1mM concentration, *ρ*_***X***_(*n*_1_,…,*n*_*s*_) is largely dominated by the empty state probability *ρ*_***X***_(0_1_,…,0_*s*_) indicating only a small fraction of bound states with non-negligible occurrences (S4 Fig). Within this fraction, the most likely states involve single occupancy of the S4S5 linker or the S4Pore interface as shown by the marginal probabilities *ρ*_***X***_(*n*_*j*_) of individual sites (Fig 2). At higher concentrations, there is a clear shift of *ρ*_***X***_(*n*_1_,…,*n*_*s*_) towards channel occupancy states that significantly enhance the average number of bound ligands. Careful inspection of *ρ*_***X***_(*n*_*j*_) confirms the major relevance of sites at the S4S5 linker and S4Pore interface over the entire concentration range, accompanied by an increasing importance of binding regions at the S6P-helix interface. In contrast, *ρ*_***X***_(*n*_*j*_) for sites within the voltage-sensor, central cavity and nearby the extracellular face of the channel remain negligible over all concentrations. For completeness, note in S1 Table that equilibrium constants for doubly-occupied sites are comparable to or even higher than estimates for one-bound molecule thus revealing important saturation effects in which one or two sevoflurane molecules can stably bind the channel structures at individual sites. The result is especially true for spots at the S4S5 linker and S4Pore interface.

**Fig 3:**
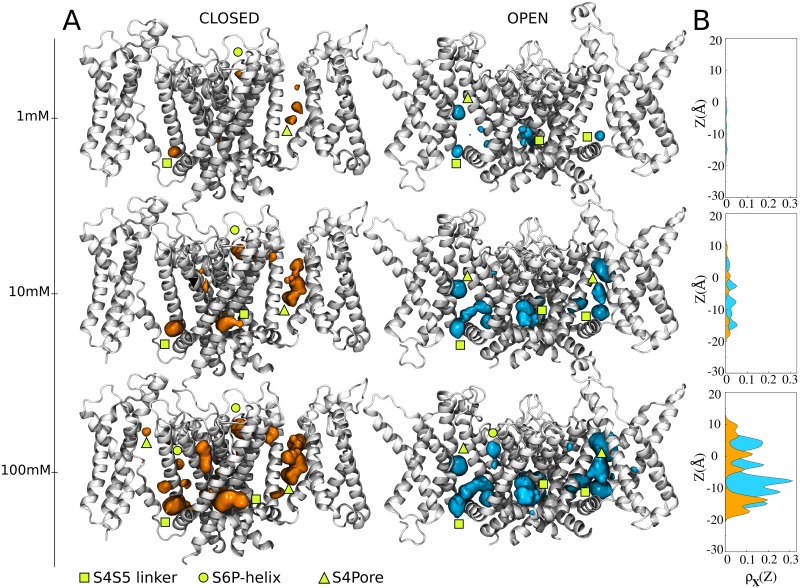
C and O position-dependent binding probabilities for diluted concentrations of sevoflurane in the bulk. (A) Shown is the ensemble average structure of the channel (white) along with the density $\rho_{X}^{j}(R)$ of sevoflurane (orange and cyan) in each of the binding sites (isovalues of 9x10^-5^ Å^-3^). Densities pertaining to sites S4S5 linker, S6P-helix and S4Pore are indicated with yellow symbols. As described in Computational Methods, the determination of $\rho_{X}^{j}(R)$ involved reweighing the marginal probability *ρ*_***X***_(*n*_*j*_) at the binding site *j* by the local equilibrium density of sevoflurane *ρ*_***X***_(***R|****n*_*j*_). The marginal *ρ*_***X***_(*n*_*j*_) was computed by coarse-graining over state probabilities in S2 Fig whereas, *ρ*_***X***_(***R|****n*_*j*_) was calculated from the centroid distributions of docking solutions shown in Fig 1B and 1C. (B) Projection of $\rho_{X}^{j}(R)$ along the transmembrane direction *z* of the system, $\rho_{X}^{j}(z)$.

The complex distributions of the multiple occupied states of structures ***X*** ≡ {***C***, ***O***} were described in three dimensions by mapping *ρ*_***X***_(*n*_1_,…,*n*_*s*_) into the position-dependent density $\rho_{X}^{j}(R)$ of sevoflurane in each binding site *j* (*cf*. Computational Methods for details). As shown in Fig 3 and supplementary S1 and S2 Movies, the density of sevoflurane better convey the results by showing the spatially-mapped concentration dependent population of bound ligands. Projection of $\rho_{X}^{j}(R)$ along the transmembrane direction *z* of the system, $\rho_{X}^{j}(z)$, stresses further the results. Note from $\rho_{X}^{j}(R)$ that sevoflurane binds channel structures in a concentration dependent manner, binding preferentially the S4S5 linker and the interfaces S4Pore and S6P-helix over a range of concentrations.

So far, our calculations demonstrate that sevoflurane binds Kv1.2 structures over a spectrum of concentrations, preferentially at the linker S4S5 and at the segment interfaces S4Pore and S6P-helix. From a physical-chemical point of view, spots at these channel regions are primarily dehydrated, lipid accessible, amphiphilic pockets providing with favorable interaction sites for the polar lipophilic sevoflurane molecule (S5 Fig). It is worth mentioning that these findings recapitulate recent photolabeling experiments demonstrating that photoactive analogs of sevoflurane do interact to the S4S5 linker and at the S6P-helix interface of the open-conductive Kv1.2 channel [22,23]. In detail, Leu317 and Thr384 were found to be protected from photoactive analogs, with the former being more protected than the latter. As shown in S6 Fig, atomic distances of bound sevoflurane to these amino-acid side chains are found here to be respectively 7.28±2.5 Å and 10.44±3.66 Å, in average more or less standard deviation. Such intermolecular distances are consistent with direct molecular interactions and therefore consistent with the measured protective reactions–similar conclusions hold for the closed channel as well. Besides that, our calculations recapitulate the stronger protection of Leu317 in the sense that, relative to sites at S6P-helix, the affinity of sevoflurane is found to be higher at the S4S5 linker considering its stable occupancy either by one or two ligands. The stable occupancy of the linker by one or two ligands as computed here, is also consistent with recent flooding-MD simulations of the homologous sodium channel NaChBac [14,24] and more importantly, with previous Ala/Val-scanning mutagenesis showing a significant impact of S4S5 mutations on the effect of general anesthetics on members of the K^+^ channel family [10]. In particular, a single residue (Gly329) at a critical pivot point between the S4S5 linker and the S5 segment underlies potentiation of Kv1.2 by sevoflurane [18]. Sevoflurane is found to be close to that amino acid when bound to the S4S5 linker.

In contrast to the aforementioned spots, sites within the voltage-sensor, within the main pore and nearby the extracellular face of the Kv1.2 structures are primarily hydrated, lipid-inaccessible, amphiphilic pockets (S5 Fig) that weaken sevoflurane interaction as reflected in the state- and space-dependent densities shown in Figs 2 and 3. The binding probabilities at these sites thus support that a non-negligible fraction of poses determined from docking (Fig 1D) corresponds to low affinity or false positives. In particular, because sevoflurane induces potentiation rather than blocking of Kv1.2 [17,18], we read the negligible or absent density of the ligand in the channel central-cavity as a self-consistent result of the study–especially for the open-conductive state. Supporting that conclusion, note that binding constants as computed here are upper bounds for the affinity of sevoflurane under ionic flux conditions in which potentiation takes place. Accordingly, as shown in S7 Fig, the binding affinity of a potassium ion at the central cavity overcomes that of sevoflurane due its binding and excess free-energies under applied voltages. Once bound, the ion destabilizes sevoflurane interactions and the molecule is not expected to bind the channel cavity at low concentrations. As also shown in S7 Fig and supplementary S3 Movie, even under the occurrence of rare binding events, sevoflurane appears unable to block the instantaneous conduction of potassium which is also consistent with its potentiating action.

Weak interactions at the main pore and nearby the selectivity filter of Kv1.2 contrasts with sevoflurane binding at analogous regions of NaChBac [14,24], likely due major structural differences between Na^+^ and K^+^ channels. Specifically, the pore of potassium channels lacks lipid-accessible open-fenestrations of the sodium relatives and K^+^-selective filters are sharply distinct from Na^+^-selective ones.

### Anesthetic binding impacts channel energetics

Despite a comparable pattern of molecular interactions, careful inspection of *ρ*_***X***_(*n*_*j*_) or $\rho_{X}^{j}(R)$ reveals that for most sites there is an obvious differential affinity of sevoflurane across Kv1.2 structures (Figs 2 and 3). The overall consequence for sevoflurane binding is then clear: the average number of ligands bound to the open-conductive channel systematically exceeds that for the resting-closed channel over the entire concentration range. There is therefore a remarkable conformational dependence for the anesthetic interaction, such that sevoflurane preferentially binds the open-conductive structure.

**Fig 4:**
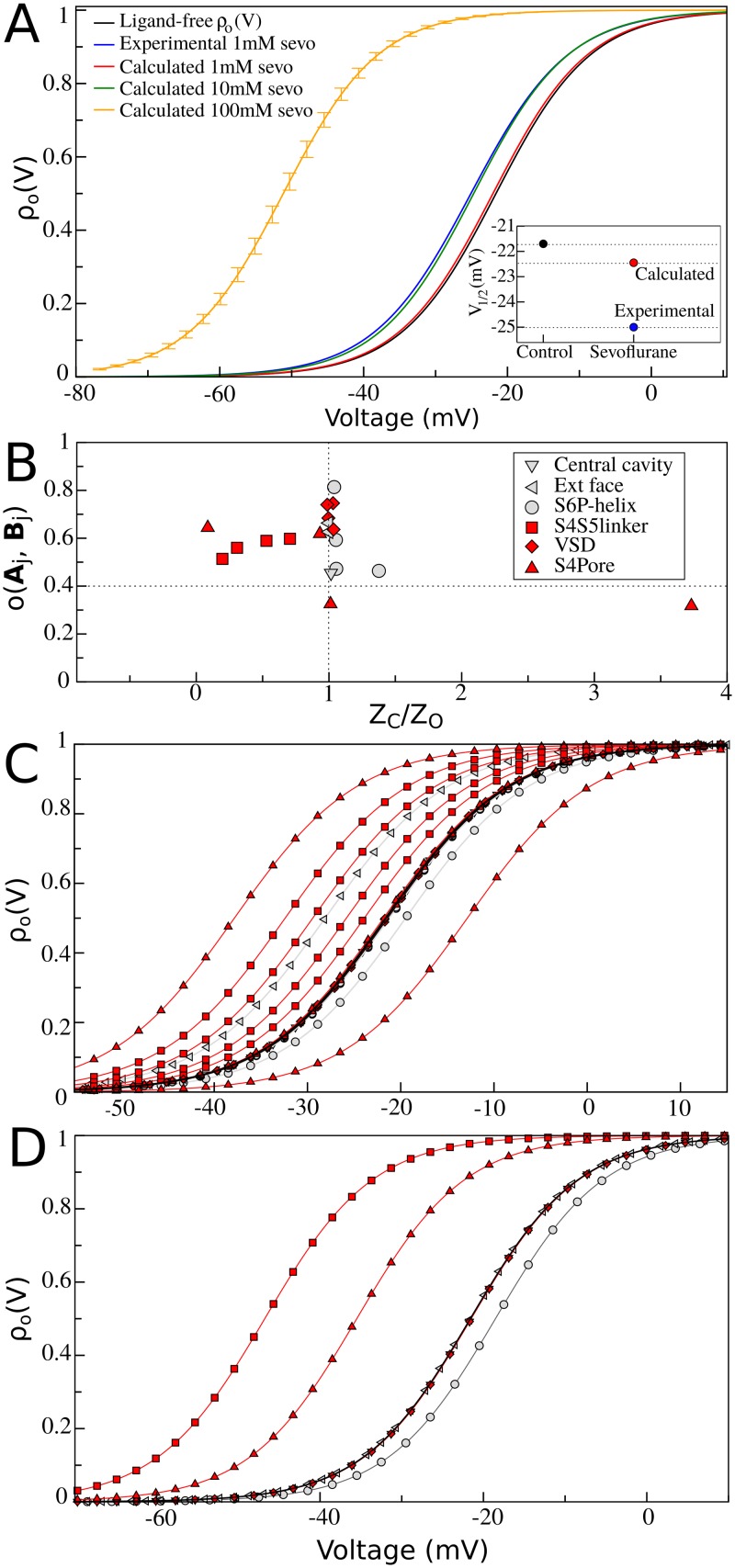
Sevoflurane binding effects on C-O equilibrium. (A) Kv1.2 open probabilities for different sevoflurane concentrations in solution. Ligand-free and ligand-bound *ρ*_*O*_(*V*) curves were respectively computed from eqs (5) and (9) by taking into consideration parameters, *V*_*m*_ = −21.9*mV* and *ΔQ* = 3.85*e*_*o*_, for best two-state Boltzmann fit of measured data for Kv1.2 free of ligands [18]. A reference experimental curve (blue) is shown for sevoflurane at 1 mM concentration, with best two-state Boltzmann parameters *V*_*m*_ = −25.1*mV* and *ΔQ* = 4.00*e*_*o*_ [18]. Relative to the ligand-free channel, 1 mM of sevoflurane shifts the open probability of the channel leftward by *ΔV*_*m*_ ≈ −3*mV*. The inset explicitly shows both experimental (blue) and calculated (red) *V*_*m*_ shifts at 1mM of sevoflurane in solution. In black, ligand-free *V*_*m*_ is also shown for reference. For sevoflurane concentrations of 1, 10 and 100 mM, their respective open probabilities *V*_*m*_ are -22.3, -24.7 and -51.1 mV. Representative statistical errors for *ρ*_*O*_(*V*) at 100mM sevoflurane concentration (error bars) were calculated by Monte Carlo bootstrap error analysis of the statistical uncertainty of independent FEP estimates considered in the calculations. (B) Decomposition analysis at 100mM ligand concentration. Shown is the FEP sampling overlap versus ratio values for **C**-**O** partition functions at the individual binding sites *j*. Per-site ratio values can be equal, smaller or larger than unity meaning respectively that sevoflurane binding is not conformational dependent, stabilizes the open structure or stabilizes the closed structure. Binding sites located nearby flexible protein regions for which the root-mean-square deviation (RMSD) between channel structures is larger than 4.0 Å are highlighted in red (*cf*. Computational Methods and S2 Fig for details). (C) Decomposition analysis of *ρ*_***O***_(*V*) curves in terms of partition ratio values showing in (B). (D) Same decomposition analysis in terms of an aggregate per-site contribution across channels subunits. At 100mM, binding of sevoflurane at the S4S5 linker and S4Pore interface significantly stabilizes the open structure of the channel which contrasts the mild stabilization of the closed structure due to ligand binding at the S6P-helix interface.

Implications for Kv1.2 energetics were then investigated by quantifying changes to the channel open probability *ρ*_***O***_(*V*) induced by sevoflurane at concentrations of 1mM– 100mM (Fig 4). Specifically, from the partition functions *Z*_***C***_(*n*_1_,…,*n*_*s*_) and *Z*_***O***_(*n*_1_,…,*n*_*s*_) across the entire ensemble of occupancy states of the channel, solution of eqs (5) and (9) show that sevoflurane shifts leftwards the open probability of Kv1.2 in a concentration-dependent manner–voltage shifts amount from -1.0 mV to -30.0 mV with concentration increase of the ligand in solution. The result is particularly interesting, supporting that the approximately 3 mV probability shift recorded experimentally at 1 mM sevoflurane concentration can be explained by our structure-based probability predictions in the concentration range of 1–10 mM. The latter thus provides a theoretical basis to predict sevoflurane impact on channel energetics in a larger, not yet experimentally probed, range of concentrations. Additionally, for a fixed ligand concentration (100 mM), decomposition analysis reveals further that ratio values for the partition functions at individual sites *j* can be smaller, equal or larger than unity, implying a non-trivial interplay of conformation-dependent modes of action involving distinct sites (*cf*. Computational Methods for details). In detail, binding of sevoflurane at low affinity sites within the voltage-sensor, central cavity and next to the extracellular face of the channel are mostly conformation-independent and do not impact open probability (ratio ≈ 1). On the other hand, conformation-dependent binding of sevoflurane to sites at the S4S5 linker and the S4Pore interface accounts for the overall stabilization of the open channel (ratio < 1). That effect contrasts with the mild stabilization of the closed conformation of Kv1.2 induced by binding of sevoflurane at S6P-helix and reflected in rightward shifts of *ρ*_***O***_(*V*) (ratio > 1). The overall conformation-dependent binding process is therefore differentially encoded across distinct channel regions.

As extensively discussed in past publications [3,17,18], potentiation of Kv1.2 by sevoflurane has been attributed to stabilization of the open-conductive state of the channel via at least two molecular mechanisms. One mechanism (i) likely involving sites allosterically coupled to the electromechanical transduction responsible for controlling voltage-dependent gating; and another (ii) implicating distinct sites, which could influence the pore conductive state stability and/or unitary conductance. Here, our structural calculations based on the open-activated and resting-closed states of Kv1.2 were consistently designed to investigate the first (i) of these mechanisms. Given the critical role of S4 and S4S5 linker on the channel gating mechanism [19], it is reasonable that sevoflurane interactions with these segments, as found here, are at the origins of the experimentally measured voltage-dependent component of anesthetic action. While restricted to sevoflurane interactions with the resting-closed and open-conductive structures, the presented two-state binding model only embodies left or rightward shifts in the open probability of the channel, therefore it cannot clarify any molecular process accounting for the maximum conductance increase recorded experimentally. As supported by a recent kinetic modeling study [17], generalization of eqs (9) to include a third non-conducting open state yet structurally unknown is needed to account for such conductance effects and for that reason, the investigation of mechanism (ii) is beyond the scope of our study. We speculate however that binding of sevoflurane at the S4Pore and S6P-helix interfaces could allosterically interfere with pore domain operation, thus affecting channel’s maximum conductance. A working hypothesis also raised in the context of anesthetic action on bacterial sodium channels [12,14], assumes indeed that non-conducting states of the selectivity filter are implicated. Corroboration of such an assumption from a molecular perspective is however not trivial and will necessarily involve further structural studies to demonstrate how ligand binding might impact non-conducting open states of the channel to affect maximum conductance.

## Discussion

Here, we carried out extensive structure-based calculations to study conformation-dependent binding of sevoflurane to multiple saturable sites of Kv1.2 structures ***X*** ≡{***C***, ***O***} under equilibrium conditions–the total MD simulation time was ~2.0 μs. Binding of sevoflurane was studied for ligand concentrations in the range of 1mM–100mM and saturation conditions up to $n_{j}^{max}=2$. Our study relied on the assumption that molecular docking calculations performed in vacuum can faithfully describe ligand interactions at protein sites. Specifically related to that assumption, we have considered the generated ensemble of docking solutions to estimate the location of binding sites *δV*_*j*_ and the local distribution of the ligand *ρ*_***X***_(***R***|*n*_*j*_). The generation of false positive hits is however a well documented drawback of docking algorithms as a result of limitations of the scoring function in describing ligand solvation energies and protein flexibility [25]. Given the same limitations of the scoring function, it is also not guaranteed that neither all binding hits, nor that *ρ*_***X***_(***R***|*n*_*j*_) can be accurately known from docking. In this regard, although not considered here, it might be important to integrate docking results from different algorithms involving different scoring functions in order to characterize the bound ensemble. Still, thanks to the generality of the presented formulation, extension of the current investigation to sampling techniques other than docking, including all-atom flooding-MD simulations [9,11,12,14,16], might also be an important refinement in that direction.

Despite these sampling improvements that may eventually be obtained, the presented combination of extensive docking calculations against an ensemble of equilibrium receptor structures fit to handle protein flexibility, and FEP calculations based on fine force-fields to accurately estimate solvation energies are critical technical aspects of the applied methodology devised to minimize such drawbacks. Whereas docking was performed in vacuum, FEP calculations were carried out in presence of explicit all-atom lipids and water, therefore taking into consideration environmental effects in the estimated ligand binding free energies. In this regard, standard binding free-energies estimated from FEP (S1 and S2 Tables) are comparable or significantly smaller than the standard free energy for transferring the ligand from water to a pure lipid bilayer–supporting that sevoflurane is expected to partition preferentially into channel sites rather than the membrane. The partition coefficient (log K) and the related transfer free energy of sevoflurane between water and the lipid bilayer (POPC) amount respectively to 2.64±0.96 and –3.12±0.32 kcal.mol^-1^ as recently estimated by Tajkhorshid and coworkers [26]. Besides that, it is also important to note that the configuration space in FEP calculations overlap between channel structures at individual sites, i.e. sampling and binding affinities were evenly resolved between states (S2 Fig and Fig 4B)–meaning eventual biases or systematic errors were mitigated when comparing similar calculations between channel states according to the main goal here.

Under these technical considerations, we conclude that most of the identified binding sites are located nearby flexible protein regions for which the root-mean-square deviation between channel structures is larger than 4.0 Å. Then for the purpose of quantifying any direct ligand effect on channel energetics, the determined conformational dependence of sevoflurane binding to these gating-implicated protein regions appears robust and likely to impact function. Structural knowledge allied to solid electrophysiological data available for Kv1.2 make this channel an interesting model system for molecular-level studies of anesthetic action thereby justifying our choice. In detail, the atomistic structures account for most of the available experimental data characterizing closed and open conformations of the channel in the native membrane environment [20]. Previous findings support further that sevoflurane binds Kv1.2 to shift leftward its voltage-dependence and to increase its maximum conductance in a dose-dependent manner [18]. Despite a similar pattern of interactions, we found here a clear conformational dependence for sevoflurane binding at multiple channel sites. The ligand binds preferentially the open-conductive structure to impact the **C**-**O** energetics in a dose-dependent manner as dictated by the classical equilibrium theory for chemical reactions embodied in eq (9). Front of the difficulty in conceiving and characterizing other, still more complex molecular processes that might impact channel energetics under applied anesthetics [5–7], the result is revealing by showing that in principle the isolated process of sevoflurane binding to Kv1.2 accounts for open-probability shifts as recorded in experiments. Within this scenario, the calculations reveal contrasting per-site contributions to the overall open probability of the channel. For instance, at 100mM concentration, binding of sevoflurane at the S4S5 linker and S4Pore interface significantly stabilizes the open structure of the channel overcoming the mild stabilization of the closed structure by ligand binding at the S6P-helix interface. By showing this non-trivial interplay of conformation-dependent modes of action involving distinct binding sites, the result is particularly insightful and should guide us to design novel site-specific mutagenesis and photolabeling experiments for further molecular characterization of anesthetic action.

Although not addressing the paucity of *in vivo* experimental evidences that a binding process to a specific molecular target as presented here is related to any clinically-relevant anesthetic outcome, our study adds support to the direct-site hypothesis by linking binding free-energy and protein energetics. As such, our study treats and reveals a new layer of complexity in the anesthetic problem that brings us novel paradigms to think their molecular action and to design/interpret research accordingly. To the best of our knowledge, the main-text Figs 3 and 4 represent in the context of structural studies, a deeper and first revealed view on the intricate mode of interactions that might take place between general anesthetics and ion channels to impact function in general.

## Methods

### Theory

#### Anesthetic binding and channel energetics

Consider the voltage-gated channel embedded in a *large* membrane-aqueous volume that contains *N* ligand molecules under dilution. The protein is assumed to remain in a well-defined conformational state ***X***, in which it presents *s* distinct binding sites for ligands. For simplicity, we consider that ligands dissolve uniformly across the membrane-aqueous region of the system from where they can partition into the protein sites. The lipid and aqueous phases thus provide with a bulk volume *V* occupied by ligands at constant density $\bar{\rho }$ and *excess* chemical potential $\bar{\mu }$. We consider further that every site *j* = 1,…,*s* corresponds to a discrete volume *δV*_*j*_ that can be populated by $0⩽n_{j}⩽n_{j}^{max}$ ligands. We denote by $O_{X}^{*}(n_{1},\ldots ,n_{s})$ the specific occupancy state featuring *n*_*j*_ bound ligands at corresponding sites and by *n* = *n*_1_+…+*n*_*s*_ the total number of bound ligands in this state.

Under these considerations, solution of ligand binding to multiple receptor sites relies fundamentally in determining the equilibrium constant *K*_***X***_(*n*_1_,…,*n*_*s*_) for the process $O_{X}^{*}(0_{1},\ldots ,0_{s})+nL\Leftrightarrow O_{X}^{*}(n_{1},\ldots ,n_{s})$ where, $O_{X}^{*}(0_{1},\ldots ,0_{s})$ is the empty receptor state with all ligands occupying the bulk. As shown in previous work [21], at a fixed temperature *β* = (*k*_*B*_*T*)^−1^, the binding constant *K*_***X***_(*n*_1_,…,*n*_*s*_) can be evaluated from MD-based free-energy perturbation (FEP) calculations

$$K_{X}(n_{1},\ldots ,n_{s})=\frac{1}{n_{1}!\ldots n_{s}!}\left[\prod_{i=1}^{n}{\left(\frac{2\pi }{\beta k_{X}(i)}\right)}^{\frac{3}{2}}\right]e^{-\beta [W_{X}^{*}(n)-n\bar{\mu }]}$$ (1)

in which $\bar{\mu }$ is the solvation free energy of the ligand in the bulk and $W_{X}^{*}(n)$ corresponds to the free-energy of *n* site-specific bound ligands relative to a gas phase state given that ligands *i* = 1,…,*n* are restrained with force constants *k*_***X***_(*i*) to occupy an *effective* site volume $\left[\prod_{i=1}^{n}{\left(\frac{2\pi }{\beta k_{X}(i)}\right)}^{\frac{3}{2}}\right]$ at structure ***X***. Eq (1) is solved for the thermodynamic limit *N* ≫ *n* and $\frac{1}{n_{1}!\ldots n_{s}!}$ corrects the binding constant for equivalent configurations of *n*_*j*_ indistinguishable ligands within the site volumes *δV*_*j*_. Within this formulation, knowledge of *K*_***X***_(*n*_1_,…,*n*_*s*_) ensures the probability of any occupancy state

$$\rho_{X}(n_{1},\ldots ,n_{s})=\frac{{\bar{\rho }}^{\left(n_{1}+\cdots +n_{s}\right)}{Κ}_{X}(n_{1},\ldots ,n_{s})}{\sum_{n_{1}^{\prime },\ldots ,n_{s}^{\prime }}{\bar{\rho }}^{\left(n_{1}^{\prime }+\cdots +n_{s}^{\prime }\right)}{Κ}_{X}(n_{1}^{\prime },\ldots ,n_{s}^{\prime })}$$ (2)

to be known in practice from free-energy calculations [27]. Here, the normalization condition appearing in the denominator of eq (2) runs over every occupancy state $O(n_{1}^{\prime },\ldots ,n_{s}^{\prime })$ of the channel, ranging from *O*(0_1_,…,0_*s*_) up to $O(n_{1}^{max},\ldots ,n_{s}^{max})$. Note in eq (2) *ρ*_***X***_(*n*_1_,…,*n*_*s*_) depends on the number density or concentration of the ligand at the reservoir thus providing a useful equation for investigation of concentration effects.

To investigate any conformational dependence on ligand binding, we consider eq (2) in the context of conformational equilibrium of the channel over a range of TM voltages. Specifically, we assume the very same microscopic system submitted to a Nernst potential induced by non-symmetrical electrolytes between membrane faces. The capacitive nature of the channel-membrane system ensures the Nernst potential accounts for a voltage difference *V* across the lipid bilayer. Accordingly, by denoting the entire set of instantaneous Cartesian coordinates of the channel as ***r***^*P*^, the free energy of the protein *F*_***X***_(*V*) in a particular conformation ***X*** ≡ ***X***(***r***^*P*^)

$$e^{-\beta F_{X}(V)}\propto \int dr^{P}\delta [X^{\prime }(r^{P})-X]e^{-\beta [U(r^{P})+Q(r^{P})V]}$$ (3)

can be written within an arbitrary constant, in terms of an effective potential energy of the protein *U*(***r***^*P*^) + *Q*(***r***^*P*^)*V* when coupled to the external voltage *V* with charge *Q*(***r***^*P*^) [28]. Note the integral runs over the entire configurational space accessible to ***r***^*P*^, so long as it is compatible with conformation ***X***, as indicated by the delta notation *δ*[***X***′(***r***^*P*^) − ***X***]. From eq (3), the open probability of the channel then reduces to

$$\rho_{O}(V)=\frac{e^{-\beta F_{O}(V)}}{e^{-\beta F_{C}(V)}+e^{-\beta F_{O}(V)}}$$ (4)

for the case of a voltage-gated channel with two conformational states ***X*** ≡ {***C***, ***O***} connected by the reaction process $C\overset{V}{\Leftrightarrow }O$. In terms of *chemical* free-energies of the receptor *F*_***C***_(*V* = 0) and *F*_***O***_(*V* = 0) and the corresponding *excess* free-energies *ΔF*_***C***_(*V*) and *ΔF*_***O***_(*V*), eq (4) simplifies into the familiar two-state Boltzmann equation

$$\rho_{O}(V)={\left[1+e^{+\beta \Delta Q(V_{m}-V)}\right]}^{-1}$$ (5)

in which,

$$\Delta Q=-\frac{\Delta F_{O}(V)-\Delta F_{C}(V)}{V}$$ (6)

is the gating charge *ΔQ* = *Q*_*O*_ − *Q*_***C***_ resulting from differences in the effective protein charge in each conformational state and

$$V_{m}=\frac{\left[F_{O}(V=0)-F_{C}(V=0)\right]}{\Delta Q}$$ (7)

is the midpoint voltage in which *ρ*_***C***_(V) = *ρ*_***O***_(*V*) [28]. From eq (5), the equilibrium constant between protein states ***C*** and ***O*** then writes as

$$Κ(V)=e^{-\beta \Delta Q[V_{m}-V]}$$

with $Κ(0)=e^{-\beta V_{m}\Delta Q}$ determining their equilibrium at 0 mV. In eqs (6) and (7), the voltage-independent free energies account for the microscopic potential energy of the channel and its solvation energy in each state whereas the corresponding voltage-dependent *excess* free energies are proportional to the applied voltage and associated protein charges.

By combining eqs (2) and (5) through a generalized thermodynamic-cycle analysis dealing with all possible states of the ligand-free and ligand-bound receptor, binding effects on the channel energetics can be then explicitly expressed over a range of membrane voltages

$$\rho_{O}(V)=\frac{Κ(V)Z_{O}(n_{1},\ldots ,n_{s})}{Z_{C}(n_{1},\ldots ,n_{s})+Κ(V)Z_{O}(n_{1},\ldots ,n_{s})}$$ (8)

in terms of the partition functions

{ZC(n1,…,ns)=∑n1′,…,ns′ρ¯(n1′+…+ns′)ΚC(n1′,…,ns′)ZO(n1,…,ns)=∑n1′,…,ns′ρ¯(n1′+…+ns′)ΚO(n1′,…,ns′)

for the ensemble of occupancy states in each protein conformation. Eq (8) simplifies into

$$\rho_{O}(V)={\left[1+\frac{Z_{C}(n_{1,}\ldots ,n_{s})}{Z_{O}(n_{1,}\ldots ,n_{s})}e^{+\beta \Delta Q[V_{m}-V)}\right]}^{-1}$$ (9)

the two-state Boltzmann equation now embodying the free-energy contributions arising from ligand binding (*cf*. Computational Methods for details).

In this contribution, we consider eqs (1), (5) and (9) to investigate the molecular binding of sevoflurane to open and closed structures of Kv1.2, and its functional impact on channel energetics.

### Computational methods

A procedure was designed to solve the molecular binding of sevoflurane to the open-conductive (**O**) and resting-closed (**C**) structures of Kv1.2 for saturation conditions up to $n_{j}^{max}=2$, under equilibrium conditions. For both channel structures, the procedure consisted of (i) an extensive production of docking solutions for the ligand-receptor interaction, (ii) clustering of docking solutions into binding sites along the receptor structure and (iii) estimation of binding affinities using the free-energy perturbation (FEP) method. First completion of steps (i) through (iii) solved the ligand channel interaction for singly-occupied binding sites. Double occupancy of the receptor sites was investigated by inputting the first generated ensemble of docked structures into another round of (i) through (iii) calculations. In detail, step (i) was accomplished by docking sevoflurane as a flexible ligand molecule against an MD-generated ensemble of membrane-equilibrated structures of the protein receptor. Docking calculations included the transmembrane domain of the channel, free from the membrane surroundings. Step (ii) provided the location of *δV*_*j*_ volumes lodging docking solutions for the ligand along the channel structures. Each of these volumes was treated as binding site regions in FEP calculations. Here, FEP calculations started from equilibrium structures of the ligand-bound channel embedded in a explicit water-membrane environment. For the purpose of improving statistics, FEP estimates and the associated statistical errors were determined from at least two independent decoupling runs. Calculations were performed over ~ 7.0 ns per site, per conformation and per replica to converge FEP estimates, in a total MD simulation time of ~ 2.0 μs. Following this procedure, binding constants *K*_***X***_(*n*_1_,…,*n*_*s*_) for channel structures ***X*** ≡ {***C***, ***O***} were solved by inputting FEP estimates into eq (1), allowing for direct solution of state-dependent probability distributions *ρ*_***X***_(*n*_1_,…,*n*_*s*_) via eq (2). Here, binding constants *K*_***X***_(*n*_1_,…,*n*_*s*_) and the related standard binding free energies $\Delta G_{X}^{o}(n_{1},\ldots ,n_{s})$ were solved for the condition of independent binding sites and relative to a homogeneous and diluted aqueous solution occupied by ligands at constant density $\bar{\rho }$ and excess chemical potential $\bar{\mu }$. Ligand-free and ligand-bound open probability curves *ρ*_***X***_(*V*) were respectively computed from eqs (5) and (9) by taking into consideration the previously determined mid-point voltage and steepness of the open probability curve of Kv1.2 free of ligands, *i*.*e*. *V*_*m*_ = −21.9 *mV* and *ΔQ* = 3.85*e*_*o*_ as determined from best two-state Boltzmann fit of the measured conductance-voltage data of the channel [18]. Note that any ligand-induced shift in eq (9) is determined by the partition function ratio between open and closed structures and not by the choice of these reference parameters. Probabilities *ρ*_***X***_(*n*_1_,…,*n*_*s*_) and *ρ*_***X***_(*V*) were determined for sevoflurane concentrations in the range of 1mM–100mM (or in density units, 6.02x10^-7^Å^-3^–6.02x10^-5^Å^-3^).

#### Membrane equilibrated channel structures

The Kv1.2 structure in the open-conductive (**O**) state was obtained from Treptow and Tarek [29]. The construct was previously acquired via molecular dynamics (MD) simulations of the published x-ray crystal structure [19]. The resting-closed (**C**) structure of Kv1.2 was obtained from Delemotte *et al*. [30]. Modeling details and validation can be found in the original papers. It is important to clarify that besides the structure by Delemotte *et al*., other resting-state models have been proposed for Kv1.2 [31–34]. Given that these structures were proven to provide with a consensus model for the resting state of the channel [35], we focus our investigation on the former model.

Structures **C** and **O** were embedded in the lipid bilayer for Molecular Dynamics (MD) relaxation and subsequent molecular docking of sevoflurane. Specifically, each structure featuring three K^+^ ions (s4s2s0) at the selectivity filter was inserted in a fully hydrated and zwitterionic all atom palmitoyloleylphosphatidylcholine (POPC) phospholipid bilayer. After assembled, each macromolecular system was simulated over a MD simulation spanning ~ 20 ns, at constant temperature (300 K) and pressure (1 atm), neutral pH, and with no applied TM electrostatic potential. The channel structures remained stable in their starting conformations throughout the simulations. Indeed, the RMSD profile of the channel in each simulation converges to a plateau value of approximately 4.0 Å, indicative of structural stability of the constructs [29,30]. Structures sampled in the steady phase of the trajectories were considered in subsequent docking and FEP calculations.

#### Molecular Docking

We used *AutoDock Vina* [36] to dock sevoflurane against the MD-generated ensemble of channel structures **C** and **O**. Each ensemble included 120 independent channel configurations at least. Docking solutions were resolved with an exhaustiveness parameter of 200, by searching a box volume of 100 x 100 x 100 Å^3^ containing the transmembrane domain of the protein receptor. Sevoflurane was allowed to have flexible bonds for all calculations. Clustering of docking solutions was carried out following a maximum neighborhood approach.

#### Molecular dynamics

All MD simulations were carried out using the program NAMD 2.9 [37] under Periodic Boundary Conditions. Langevin dynamics and Langevin piston methods were applied to keep the temperature (300 K) and the pressure (1 atm) of the system fixed. The equations of motion were integrated using a multiple time-step algorithm [38]. Short- and long-range forces were calculated every 1 and 2 time-steps respectively, with a time step of 2.0 fs. Chemical bonds between hydrogen and heavy atoms were constrained to their equilibrium value. Long-range electrostatic forces were taken into account using the Particle Mesh Ewald (PME) approach [39]. The CHARMM36 force field [40] was applied and water molecules were described by the TIP3P model [41]. All the protein charged amino acids were simulated in their full-ionized state (pH = 7.0). All MD simulations, including FEP and voltage-driven simulations (see next), were performed on local HPC facility at LBTC.

#### Free-energy perturbation (FEP)

Eq (1) was simplified here for the condition of ligand interactions to multiple independent sites–a condition that appears to be fulfilled at the channel structures featuring sparse binding sites for sevoflurane. Within this scenario, binding constants for structures ***X*** ≡ {***C***, ***O***} were factorized as the product of independent equilibrium constants

$${Κ}_{X}(n_{1},\ldots ,n_{s})={Κ}_{X}(n_{1},0_{2},\ldots ,0_{s})\times \ldots \times {Κ}_{X}(0_{1},\ldots ,0_{s-1},n_{s})$$

where,

$$\begin{array}{c} {Κ}_{X}(n_{1},0_{2},\ldots ,0_{s})=\frac{1}{n_{1}!}\left[\prod_{i=1}^{n_{1}}{\left(\frac{2\pi }{\beta k_{X}(i)}\right)}^{\frac{3}{2}}\right]e^{-\beta [W_{X}^{*}(n_{1})-n_{1}\bar{\mu }]}\\ \ldots \\ {Κ}_{X}(0_{1},\ldots ,0_{s-1},n_{s})=\frac{1}{n_{s}!}\left[\prod_{i=1}^{n_{s}}{\left(\frac{2\pi }{\beta k_{X}(i)}\right)}^{\frac{3}{2}}\right]e^{-\beta [W_{X}^{*}(n_{s})-n_{s}\bar{\mu }]} \end{array}$$

denote respectively the binding constant of *n*_*j*_ ligands to each of the *j* sites at structure ***X***.

Accordingly, the *excess* chemical potential $\bar{\mu }$ associated with coupling of the ligand from gas phase to bulk water and $W_{X}^{*}(n_{j})$ associated with coupling of *n*_*j*_ ligands from gas phase to site *j* under restraints were quantified via FEP. Because computation of $\bar{\mu }$ does not depend on the choice of concentration, so long as the same thermodynamic state is used for the solution and gas phases, we estimated the *excess* potential by considering one sevoflurane molecule embedded into a water box of 60 x 60 x 60 Å^3^. $W_{X}^{*}(n_{j})$ was computed by taking into consideration the whole ligand-channel-membrane system.

All FEP calculations were performed in NAMD 2.9 [37] by considering the Charmm-based parameters for sevoflurane as devised by Barber *et al*. [42]. Starting from channel-membrane equilibrated systems containing bound sevoflurane as resolved from docking, forward transformation were carried out by varying the coupling parameter in steps of 0.01. Each transformation then involved a total of 100 windows, each spanning over 31800 steps of simulation. For the purpose of improving statistics, free-energy estimates and associated statistical errors were determined using the simple overlap sampling (SOS) formula [43] based on at least two independent FEP runs.

Specifically for ligand-protein calculations, the free-energy change $W_{X}^{*}(1_{j})$ for singly-occupied sites *j* was computed as a FEP process that involves ligand coupling to a vacant site. Differently, for doubly-occupied sites, $W_{X}^{*}(2_{j})$ was computed as a two-step FEP process involving ligand coupling to a vacant site $W_{X}^{*}(1_{j})$ followed by binding of a second ligand at the preoccupied site $W_{X}^{*}(2_{j}|1_{j})$. Because $W_{X}^{*}(2_{j})$ is a state function, the stepwise approach is equivalent to a single-step process involving simultaneous coupling of two ligands to the protein site that is, $W_{X}^{*}(2_{j})=W_{X}^{*}(1_{j})+W_{X}^{*}(2_{j}|1_{j})$. The colvars module [44] in NAMD 2.9 was used to apply the harmonic restraint potentials when computing these quantities.

The value of $W_{X}^{*}(n_{j})$ depends on the parameters of the restraint potential adopted in the FEP calculation, *i*.*e*. the reference positions of the ligands in the bound state $\text{\{}R_{X}^{*}(1_{j}),\ldots ,R_{X}^{*}(n_{j})\text{\}}$ and the magnitude of force constants {*k*_***X***_(1_*j*_),…,*k*_***X***_(*n*_*j*_)}. By minimizing the contribution of the restraint potential to the binding free-energy $W_{X}^{*}(n_{j})$, Roux and coworkers [45] devised optimum choices for the parameters

$$\left\{R_{X}^{*}(1_{j})=\langle R_{X}(1_{j})\rangle ,\ldots ,R_{X}^{*}(n_{j})=\langle R_{X}(n_{j})\rangle \right\}$$

and

$$\left\{k_{X}(1_{j})=\frac{3\beta^{-1}}{\left\langle {\left[\delta R_{X}(1_{j})\right]}^{2}\right\rangle },\ldots ,k_{X}(n_{j})=\frac{3\beta^{-1}}{\left\langle {\left[\delta R_{X}(n_{j})\right]}^{2}\right\rangle }\right\}$$

in which, ⟨***R***_***X***_(1_*j*_)⟩,…,⟨***R***_***X***_(*n*_*j*_)⟩ and ⟨[*δ****R***_***X***_(1_*j*_)]^2^⟩,…,⟨[*δ****R***_***X***_(*n*_*j*_)]^2^⟩ are respectively the equilibrium average positions for each of the *n*_*j*_ bound ligands at site *j* and their corresponding mean-square fluctuations when interacting to structure ***X***. Here, these parameters were estimated from the space of docking solutions and the resulting force constants, in the range of 0.03 to 1.35 kcal/mol/Å^2^, were considered for computations of the bound state.

#### Sampling overlap

Here, a per-site measure of sampling overlap *o*(***A***_*j*_, ***B***_*j*_) between FEP configurations in structures **C** and **O**

$$o(A_{j},B_{j})=1-\frac{\sqrt{tr({\left({A_{j}}^{1/2}-{B_{j}}^{1/2}\right)}^{2})}}{\sqrt{trA_{j}+trB_{j}}}$$

was determined [46] from the square root of the covariance matrices ***A***_*j*_ and ***B***_*j*_ associated respectively to **C** and **O** samples at site *j*. Specifically, ***A***_*j*_ and ***B***_*j*_ were computed as symmetric 3 × 3 covariance matrices for centroid positions ***R***_*j*_ of the ligand at site *j*

$$X_{j}=\langle (R_{j}-\langle R_{j}\rangle ).{\left(R_{j}-\langle R_{j}\rangle \right)}^{T}\rangle$$

and their square roots

$$X^{1/2}=Rdiag(\lambda_{1}^{1/2},\lambda_{2}^{1/2},\lambda_{3}^{1/2})R^{T}$$

were solved from the column major eigenvectors {***R***_*l*_, ***R***_2_, ***R***_3_} of the rotation matrix ***R*** and the associated eigenvalues {***λ***_*l*_, ***λ***_2_, ***λ***_3_}. Note that overlap is expectedly 1 for identical samplings and 0 for orthogonal configuration spaces.

#### Absolute binding free energy and ensemble averages

An absolute binding free-energy $\Delta G_{X}^{o}(n_{1},\ldots ,n_{s})$ [47] associated with state $O_{X}^{*}(n_{1}^{\prime },\ldots ,n_{s}^{\prime })$ can be defined as

$$\Delta G_{X}^{o}(n_{1},\ldots ,n_{s})=-\beta^{-1}ln[K_{X}(n_{1},\ldots ,n_{s})\times {\left(C^{o}\right)}^{n}]$$

where it is understood that this refers to the free energy of binding *n* ligands to the protein structure ***X*** ≡ {***C***, ***O***} from a reference standard reservoir concentration C° = 1M or in units of number density C° = (1,660Å^3^)^−1^. Still, the ensemble average of any thermodynamic property of the system $A_{X}(n_{1}^{\prime },\ldots ,n_{s}^{\prime })$ for state $O_{X}^{*}(n_{1}^{\prime },\ldots ,n_{s}^{\prime })$ can be known

$$\langle A_{X}\rangle =\sum_{n_{1}^{\prime },\ldots ,n_{s}^{\prime }}{\left\langle A_{X}\right\rangle }_{\left(n_{1}^{\prime },\ldots ,n_{s}^{\prime }\right)}\rho_{X}(n_{1}^{\prime },\ldots ,n_{s}^{\prime })$$

from the state probability in eq (2).

#### Position-dependent probability densities

As demonstrated in reference [21], state-dependent probabilities *ρ*_***X***_(*n*_1_,…,*n*_*s*_) for channel structures ***X*** ≡ {***C***, ***O***} can be mapped into the probability density *ρ*_***X***_(***R***) of any given ligand *i* to occupy position ***R*** in the system (regardless the position of the remaining *N* − 1 ligands). Given our original consideration that the reservoir is a homogeneous volume occupied by ligands with position-independent density $\bar{\rho }$, the probability *ρ*_***X***_(***R***) simplifies to

ρX(R)={ρXj(R),∀R∈δVjρ¯,reservoir

for every protein site *j* = 1,…,*s*. The determination of *ρ*_***X***_(***R***) thus reduces in practice to knowledge of the per-site density $\rho_{X}^{j}(R)$

$$\rho_{X}^{j}(R)=\sum_{n_{j}=0}^{n_{j}^{max}}\rho_{X}(n_{j})\times \rho_{X}(R|n_{j})$$

where, *ρ*_***X***_(***R***|*n*_*j*_) is the local density at site *j* when occupied exactly by *n*_*j*_ molecules and *ρ*_***X***_(*n*_*j*_) is the probability for this occupancy state. The probability *ρ*_***X***_(***R***|*n*_*j*_) describes the local equilibrium density of the ligand, conditional to a specific number of bound molecules that satisfies $\int_{\delta V_{j}}dR{\;\rho }_{X}(R|n_{j})=n_{j}$. In contrast,

$$\rho_{X}(n_{j})=\sum_{n_{1}^{\prime },\ldots ,n_{s}^{\prime }}\delta_{n_{j}^{\prime },n_{j\;}}\rho_{X}(n_{1}^{\prime },\ldots ,n_{s}^{\prime })$$

denotes the marginal probability of site *j* to be occupied by *n*_*j*_ ligands regardless the occupancy of the other sites.

The formulation above establishes a formal relation between space-dependent and state-dependent densities of the system. At a fine level, this relation involves the set of equilibrium constants *K*_***X***_(*n*_1_,…,*n*_*s*_) satisfying *ρ*_***X***_(*n*_*j*_). From $\rho_{X}^{j}(R)$, spatial projections of *ρ*_***X***_(***R***) along the transmembrane *z* direction of the system can be determined as

$$\rho_{X}(z)=\bar{\rho }\times A(z)+\sum_{j=1}^{s}\rho_{X}^{j}(z)$$

where, *A*(*z*) = *ΔxΔy* is the total area of the membrane-aqueous region along the Cartesian *x* and *y* directions.

#### Coarse-graining over states

Consider any macrostate $O_{X}^{*}(n)$ of the system mapping an ensemble of accessible states $O_{X}^{*}(n_{1},\ldots ,n_{s})$ in which *n* ligands bind the receptor regardless their specific distributions over the binding sites. Because $O_{X}^{*}(n)$ is degenerate, the probability density of the macrostate

$$\rho_{X}(n)=\sum_{n_{1}^{\prime },\ldots ,n_{s}^{\prime }}\delta_{n^{\prime },n}\rho_{X}(n_{1}^{\prime },\ldots ,n_{s}^{\prime })$$

can be determined by coarse-graining over the receptor states $O_{X}^{*}(n_{1},\ldots ,n_{s})$ featuring exactly *n* = *n*_1_+…+*n*_*s*_ bound ligands. Here, the Kronecker delta function *δ*_*n*_′, *n* ensures summation over states accessible to $O_{X}^{*}(n)$ only.

#### Binding of potassium and sevoflurane at the main-pore of Kv1.2

FEP calculations to quantify the binding free-energy of sevoflurane against a preoccupied central cavity of Kv1.2 with bound potassium was computed as described in the Free-Energy Perturbation (FEP) section. Specifically, the free-energy change $W_{O}^{*}(2_{j})$ for double occupancy of the central-cavity by potassium and sevoflurane was computed as a two-step FEP process involving coupling of the ion to the central cavity $W_{O}^{*}(1_{j})$ followed by binding of the anesthetic at the preoccupied cavity $W_{O}^{*}(2_{j}|1_{j})$, that is, $W_{O}^{*}(2_{j})=W_{O}^{*}(1_{j})+W_{O}^{*}(2_{j}|1_{j})$. Absolute binding free energies $\Delta G_{O}^{o}(0_{1},\ldots ,1_{j},\ldots ,0_{s})$ and $\Delta G_{O}^{o}(0_{1},\ldots ,2_{j},\ldots ,0_{s})$ were then computed from the respective per-site binding constants *K*_***O***_(0_1_,…,1_*j*_,…,0_*s*_) and *K*_***O***_(0_1_,…,2_*j*_,…,0_*s*_). An in-water *excess* potential of -65.10 kcal.mol^-1^ was estimated for potassium. Specifically for K^+^, a total binding free-energy was obtained by summing up its absolute binding free energy with its charge (*q*) excess free energy (*qϕV*) under an applied external voltage *V* [48,49]. The voltage coupling *ϕ* was determined in the form of the “electrical distance”

$$\delta_{\epsilon }=\frac{\partial }{\partial V}{\Phi (V)}_{|V=0}$$

where, *Φ*(*V*) is the local-electrostatic potential of the ion at the central cavity of the open channel. In practice, we solve *δ*_*ϵ*_ from two independent 2ns-long simulations at voltages *V* = 0*mV* and *V* = 600*mV*. For both runs, *Φ*(*V*) was estimated from the electrostatic potential map of the system and subsequently considered to solve *δ*_*ϵ*_ for *δV* = 600*mV*.

To investigate the conduction properties of Kv1.2 with sevoflurane bound to the main pore, the open channel structure was simulated under depolarized-membrane conditions using a charge-imbalance protocol [50].

#### Partition function decomposition

In the limit of *s* independent sites, binding constants can be factorized as the product of independent equilibrium constants then ensuring the associated partition function to be factorized in terms

ZX(n1,…,ns)=ZX(n1,02,…,0s)×…×ZX(01,…,0s-1,ns)

of per-site contributions. That decomposition is particularly useful to estimate the per-site contributions impacting the open probability of the channel as defined in eq (9). For any given site *j*, ratio values

$$\frac{Z_{C}(0_{1},\ldots ,n_{j},\ldots ,0_{s})}{Z_{O}(0_{1},\ldots ,n_{j},\ldots ,0_{s})}\;\left\{ \begin{array}{c} =1\\ <1\\ >1 \end{array}\right.$$

mean respectively that ligand binding is not conformational dependent, stabilizes the open structure or stabilizes the closed structure.

#### Derivation of main-text Eq (8)

The voltage-dependent open probability for a two-state channel writes according to

$$\rho_{O}(V)=\frac{\rho_{O}(V)}{\rho_{C}(V)+\rho_{O}(V)}$$

where,

{ρC(V)=ρC(01,…,0s,V)+∑n1′,…,ns′≠01,…,0sρC(n1′,…,ns′)ρO(V)=ρO(01,…,0s,V)+∑n1′,…,ns′≠01,…,0sρO(n1′,…,ns′)

embodies respectively every occupancy state $O(n_{1}^{\prime },\ldots ,n_{s}^{\prime })$ of the channel conformations ***X*** ≡ {***C***, ***O***}. The state probabilities rewrite according to

$$\{\begin{array}{c} \rho_{C}\left(V\right)=\rho_{C}\left(0_{1},\ldots ,0_{s},V\right)\times \sum_{n_{1}^{\prime },\ldots ,n_{s}^{\prime }}{\bar{\rho }}^{\left(n_{1}^{\prime }+\ldots +n_{s}^{\prime }\right)}{Κ}_{C}\left(n_{1}^{\prime },\ldots ,n_{s}^{\prime }\right)\\ \rho_{O}\left(V\right)=\rho_{O}\left(0_{1},\ldots ,0_{s},V\right)\;K\left(V\right)\times \sum_{n_{1}^{\prime },\ldots ,n_{s}^{\prime }}{\bar{\rho }}^{\left(n_{1}^{\prime }+\ldots +n_{s}^{\prime }\right)}{Κ}_{O}\left(n_{1}^{\prime },\ldots ,n_{s}^{\prime }\right) \end{array}$$

by noting that the voltage-dependent equilibrium constant between channel conformations is given by

$$\rho_{O}(0_{1},\ldots ,0_{s},V)=\rho_{C}(0_{1},\ldots ,0_{s},V)Κ(V)$$

and that every occupancy probability

{ρC(n1,…,ns)=ρC(01,…,0s,V)×ρ¯(n1+…+ns)ΚC(n1,…,ns)ρO(n1,…,ns)=ρO(01,…,0s,V)×ρ¯(n1+…+ns)ΚO(n1,…,ns)

derives from the respective binding constant and voltage-dependent probability of the ligand-free reference state. From above, we then conclude that the voltage-dependent open probability of the channel can be expressed in terms of the partition functions

$$\{\begin{array}{c} Z_{C}\left(n_{1},\ldots ,n_{s}\right)=\sum_{n_{1}^{\prime },\ldots ,n_{s}^{\prime }}{\bar{\rho }}^{\left(n_{1}^{\prime }+\ldots +n_{s}^{\prime }\right)}{Κ}_{C}\left(n_{1}^{\prime },\ldots ,n_{s}^{\prime }\right)\\ Z_{O}\left(n_{1},\ldots ,n_{s}\right)=\sum_{n_{1}^{\prime },\ldots ,n_{s}^{\prime }}{\bar{\rho }}^{\left(n_{1}^{\prime }+\ldots +n_{s}^{\prime }\right)}{Κ}_{O}\left(n_{1}^{\prime },\ldots ,n_{s}^{\prime }\right) \end{array}$$

such that,

$$\rho_{O}(V)=\frac{Κ(V)Z_{O}(n_{1},\ldots ,n_{s})}{Z_{C}(n_{1},\ldots ,n_{s})+Κ(V)Z_{O}(n_{1},\ldots ,n_{s})}$$

## Supporting information

S1 Fig Root mean square deviation (RMSD) profiles of channel structures C and O along equilibrium MD simulations. Heavy TM domain atoms of the channel were included in the calculation, considering the starting conformation (simulation time t = 0 ns) as the reference structure. Channel structures remained stable throughout the simulations. RMSD profiles converge to a plateau value of approximately 4.0 Å, indicative of structural stability of the constructs. Equilibrium structures sampled in the steady phase of the trajectories were used in subsequent docking and FEP calculations.
(TIFF)

S2 FigOverlap analysis.(A) Per-site distributions of one and two sevoflurane molecules bound to channel structures **C** and **O**. Distributions were sampled in FEP calculations based on reference positions $\text{\{}R_{X}^{*}(1_{j}),R_{X}^{*}(2_{j})\text{\}}$ and force constants {*k*_***X***_(1_*j*_), *k*_***X***_(2_*j*_)} known from docking (*cf*. Computational Methods). Only centroid positions of the ligand are shown (dots). All binding sites but spots at the S6P-helix and the extracellular face of the channel are located nearby flexible protein regions (light to dark red) for which the root-mean-square deviation (RMSD) between channel structures is larger than 4.0 Å. (B) Sampling overlap *O*(*A*_*j*_, *B*_*j*_) between ligand distributions in (A) (*cf*. Computational Methods). Overlap is larger than 0.4 for the majority of biding sites implying a similar set of configurations effectively sampled for closed and open states. Here, RMSD and overlap were computed after elimination of overall structural rotation and translation by fitting channel structures at segments S1, S2, S3 and P-helix.(TIFF)

S3 Fig Re-hydration and re-lipidation. Site-specific lipid or water coordination number difference (Δn) between final FEP configuration and equilibrium trajectories. For both closed (**C**) and open (**O**) channel structures, Δn is a function of lipid or water distances from individual binding site’s geometric center. Δn is computed considering the average number of water/lipid molecules within throughout equilibrium trajectories (cf. S1 Fig), as well as the average coordination number in the same binding sites in all four channel subunits at the end of the FEP calculation. Averaging statistical uncertainty is propagated and shown as error bars. Note that coordination number at the final ligand-decoupled stage of FEP is very similar (Δn≈0) to equilibrium reference values determined for membrane-embedded, ligand-free channel structures. Note as well in S5 Fig that sites S4S5 linker and S6P-helix interface are lipid exposed, whereas sites within the voltage-sensor, S4Pore interface and extracellular face are water accessible.
(TIFF)

S4 Fig State-dependent binding probabilities for different concentrations of sevoflurane in the bulk. Shown are sorted values of *ρ*(*n*_1_,…,*n*_*s*_) over the occupancy states of channel structures **C** and **O**. Strings for the four most likely states are stated in the center of the plots–the first line corresponding to the most likely, and the last to the fourth most likely state.
(TIFF)

S5 Fig Local molecular interactions and physical chemical environment of sevoflurane within Kv1.2 binding sites. The first column of each conformation (**C** and **O**) displays a surface representation of the amino acids that compose the respective binding site, colored by their physical chemical character–white: apolar, green: polar non-charged, red: negatively charged and blue: positively charged. The second column shows the water molecules within a 5Å radius from the site’s geometrical center. Of note, sites S4S5 linker and S6P-helix are predominantly dehydrated and lipid accessible.
(TIFF)

S6 Fig Average atomic distances between the centroids of sevoflurane and photolabeled Kv1.2 residues in structures C and O. Shown are average distances between sevoflurane when bound to S6P-helix site and Thr384, and average distances of the ligand when bound to S4S5-linker site to residues Leu317 and G329. Distances were measured by considering both ensembles of equilibrium protein structures inputed into docking searches, and sevoflurane docking poses pertaining to a given binding site.
(TIFF)

S7 Fig Ion-sevoflurane equilibrium. (A) Absolute and excess free-energies (kcal.mol^-1^) for binding potassium (yellow) and/or sevoflurane (blue) at the main pore of Kv1.2 (white). Excess free energies at 100mV are shown in parentheses. More favorable absolute and excess free-energies ensure single occupancy by potassium to be more likely than that by sevoflurane. In contrast, double occupancy by potassium and sevoflurane is unfavorable due to a positive absolute free energy for binding the molecule at the ion occupied cavity. (B) Shown are time-dependent trajectories of potassium ions diffusing through the open pore of Kv1.2 despite one bound sevoflurane molecule at the central cavity. The voltage-driven MD simulations were carried out at a depolarized potential of 600 mV to increase the rate of sampling of conduction events. Simulations spanned a total of ~ 30 ns.
(TIFF)

S1 Movie Three-dimensional visualization of position-dependent densities of sevoflurane bound to the closed Kv1.2, at 100 mM. Kv1.2 channel and ligand densities are depicted in white and orange, respectively. Video displays 360° side view, followed by an extracellular view. Relates to main text Fig 3.
(MPG)

S2 Movie Three-dimensional visualization of position-dependent densities of sevoflurane bound to the open Kv1.2, at 100 mM. Kv1.2 channel and ligand densities are depicted in white and blue, respectively. Video displays 360° side view, followed by an extracellular view. Relates to main text Fig 3.
(MPG)

S3 Movie Ion conduction simulation in the presence of sevoflurane bound to channel central cavity. Molecular dynamics simulation with 600mV depolarizing potential was carried out to enhance sampling of conduction events. Kv1.2 channel, sevoflurane and K^+^ ions can be seen in white, blue and yellow, respectively. Water molecules nearby potassium ions and sevoflurane are also shown. Note that the presence of sevoflurane does not hinder ion conduction.
(MPG)

S1 Table FEP calculations and equilibrium binding constants for singly- and doubly-occupied sites *j* of the closed channel structure^#^. (PDF)

S2 Table FEP calculations and equilibrium binding constants for singly- and doubly-occupied sites *j* of the open channel structure^#^. (PDF)
