# Supplementary figures and images for: Binding of the general anesthetic sevoflurane to ion channels
Source: PLoS Comput Biol. 2018 Nov 26;14(11):e1006605. doi: 10.1371/journal.pcbi.1006605 (PMC6283617; doi:10.1371/journal.pcbi.1006605)

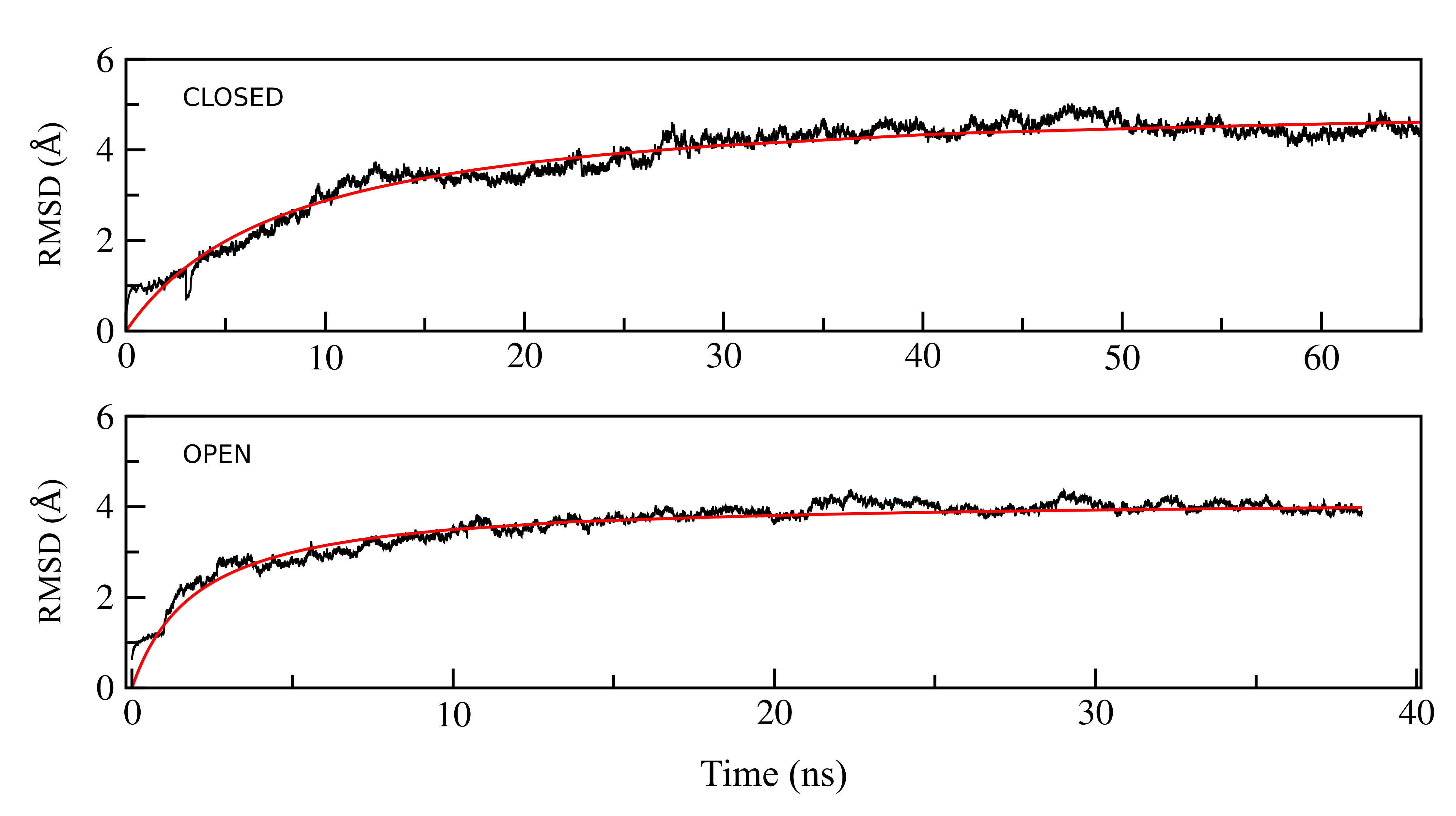

Supplement: S1 Fig — Heavy TM domain atoms of the channel were included in the calculation, considering the starting conformation (simulation time t = 0 ns) as the reference structure. Channel structures remained stable throughout the simulations. RMSD profiles converge to a plateau value of approximately 4.0 Å, indicative of structural stability of the constructs. Equilibrium structures sampled in the steady phase of the trajectories were used in subsequent docking and FEP calculations. (TIFF) [file pcbi.1006605.s001.tiff]

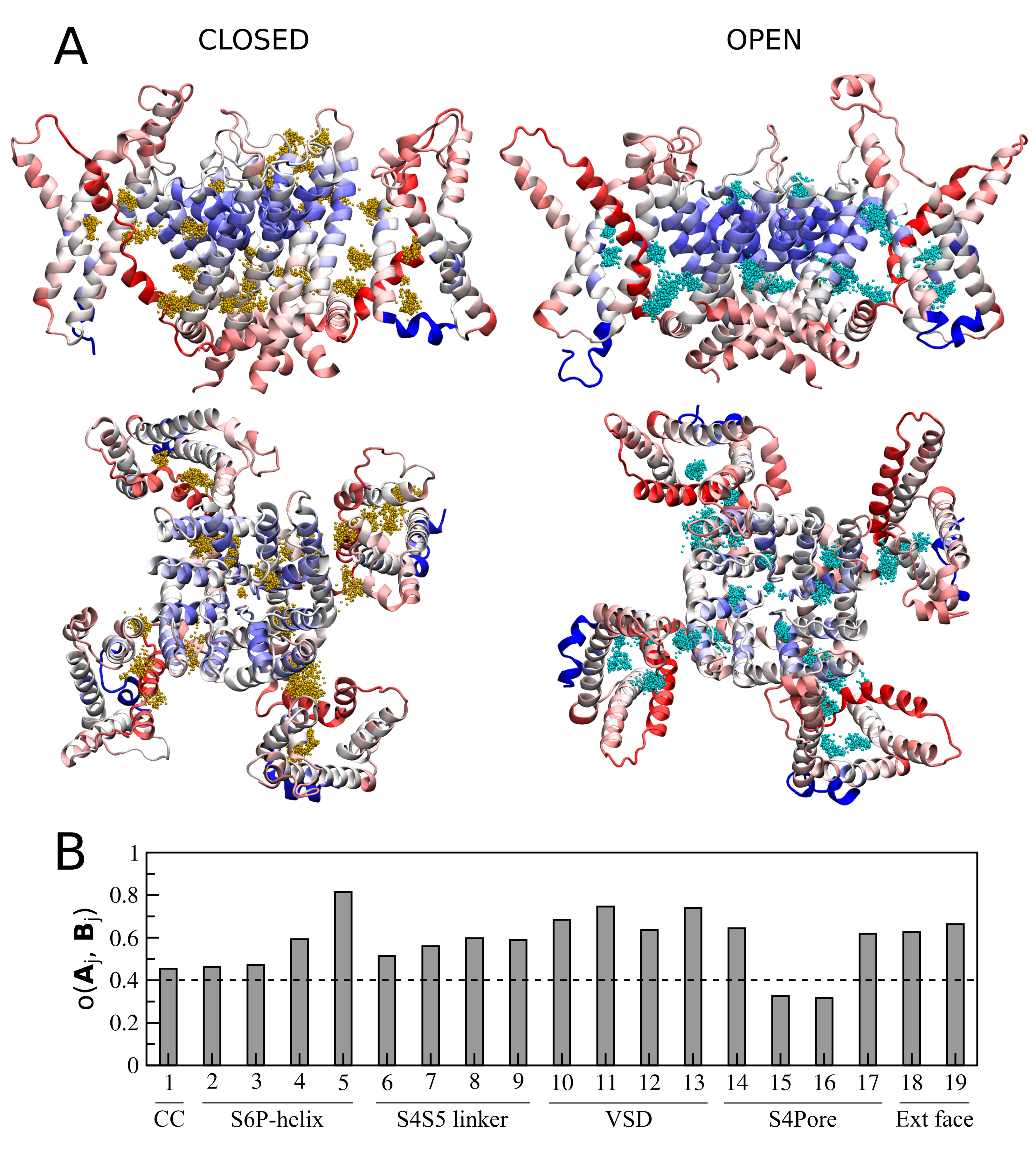

Supplement: S2 Fig — (A) Per-site distributions of one and two sevoflurane molecules bound to channel structures C and O. Distributions were sampled in FEP calculations based on reference positions {RX*(1j),RX*(2j)} and force constants {kX(1j), kX(2j)} known from docking (cf. Computational Methods). Only centroid positions of the ligand are shown (dots). All binding sites but spots at the S6P-helix and the extracellular face of the channel are located nearby flexible protein regions (light to dark red) for which the root-mean-square deviation (RMSD) between channel structures is larger than 4.0 Å. (B) Sampling overlap O(Aj, Bj) between ligand distributions in (A) (cf. Computational Methods). Overlap is larger than 0.4 for the majority of biding sites implying a similar set of configurations effectively sampled for closed and open states. Here, RMSD and overlap were computed after elimination of overall structural rotation and translation by fitting channel structures at segments S1, S2, S3 and P-helix. (TIFF) [file pcbi.1006605.s002.tiff]

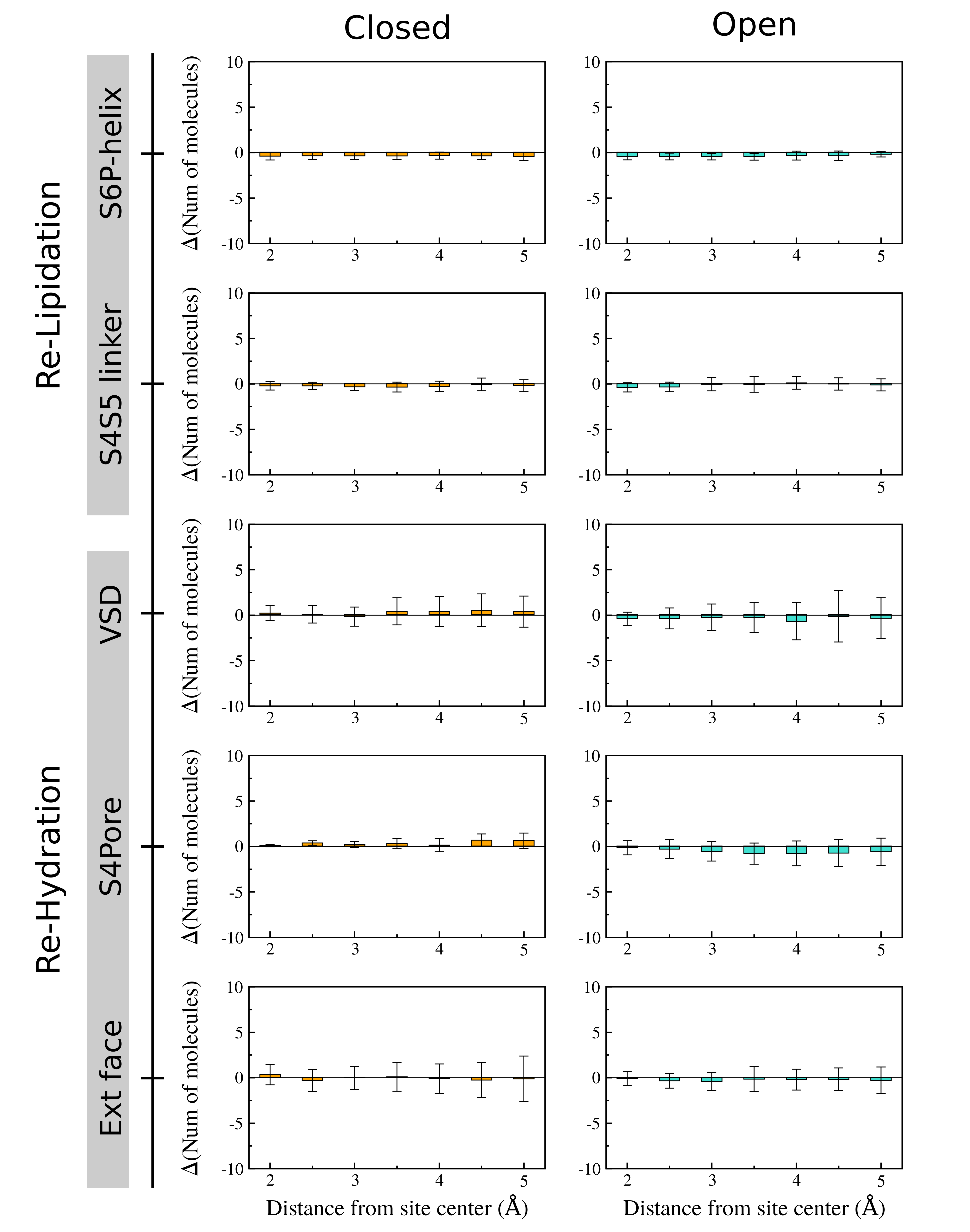

Supplement: S3 Fig — Site-specific lipid or water coordination number difference (Δn) between final FEP configuration and equilibrium trajectories. For both closed (C) and open (O) channel structures, Δn is a function of lipid or water distances from individual binding site’s geometric center. Δn is computed considering the average number of water/lipid molecules within throughout equilibrium trajectories (cf. S1 Fig), as well as the average coordination number in the same binding sites in all four channel subunits at the end of the FEP calculation. Averaging statistical uncertainty is propagated and shown as error bars. Note that coordination number at the final ligand-decoupled stage of FEP is very similar (Δn≈0) to equilibrium reference values determined for membrane-embedded, ligand-free channel structures. Note as well in S5 Fig that sites S4S5 linker and S6P-helix interface are lipid exposed, whereas sites within the voltage-sensor, S4Pore interface and extracellular face are water accessible. (TIFF) [file pcbi.1006605.s003.tiff]

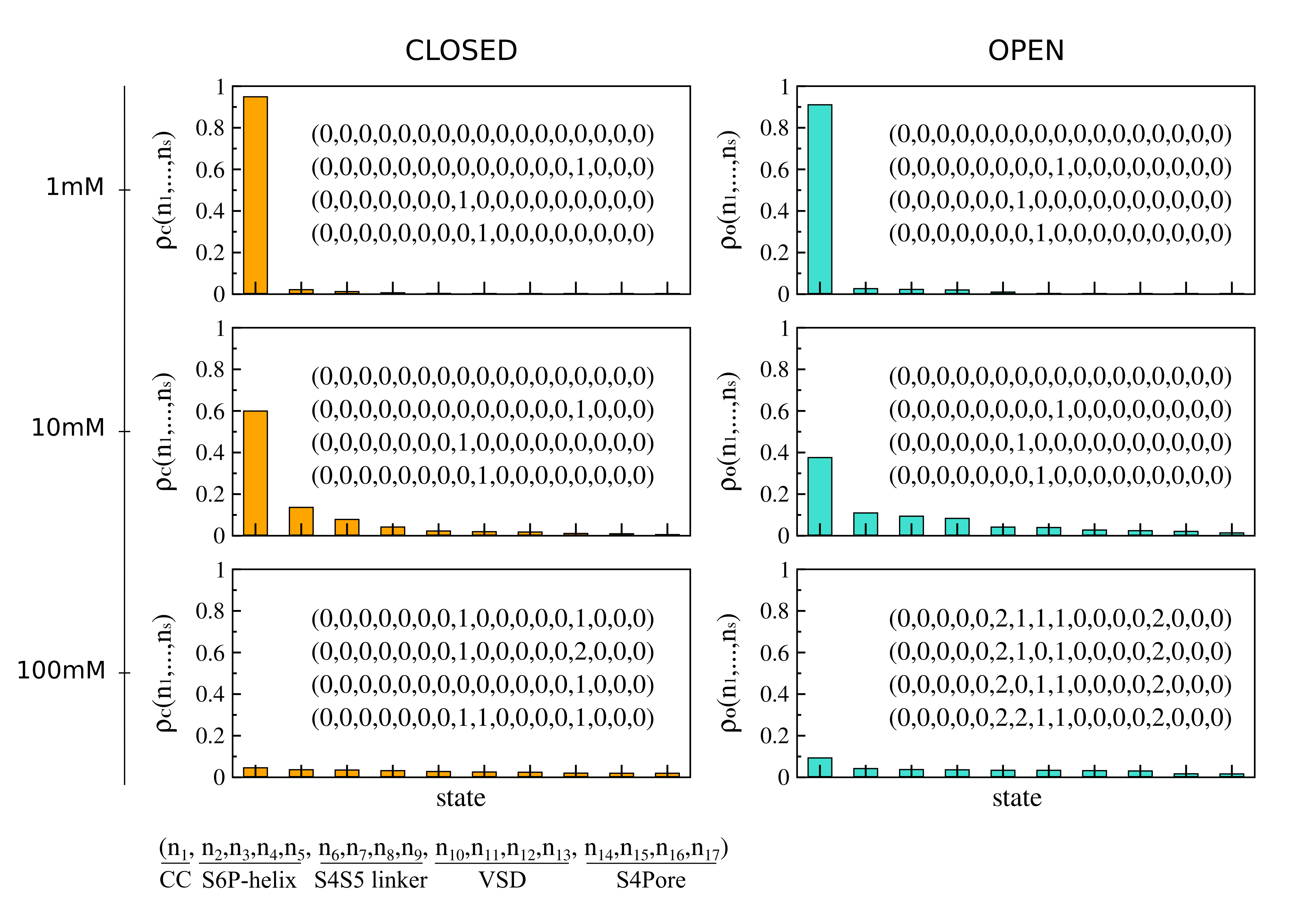

Supplement: S4 Fig — Shown are sorted values of ρ(n1,…,ns) over the occupancy states of channel structures C and O. Strings for the four most likely states are stated in the center of the plots–the first line corresponding to the most likely, and the last to the fourth most likely state. (TIFF) [file pcbi.1006605.s004.tiff]

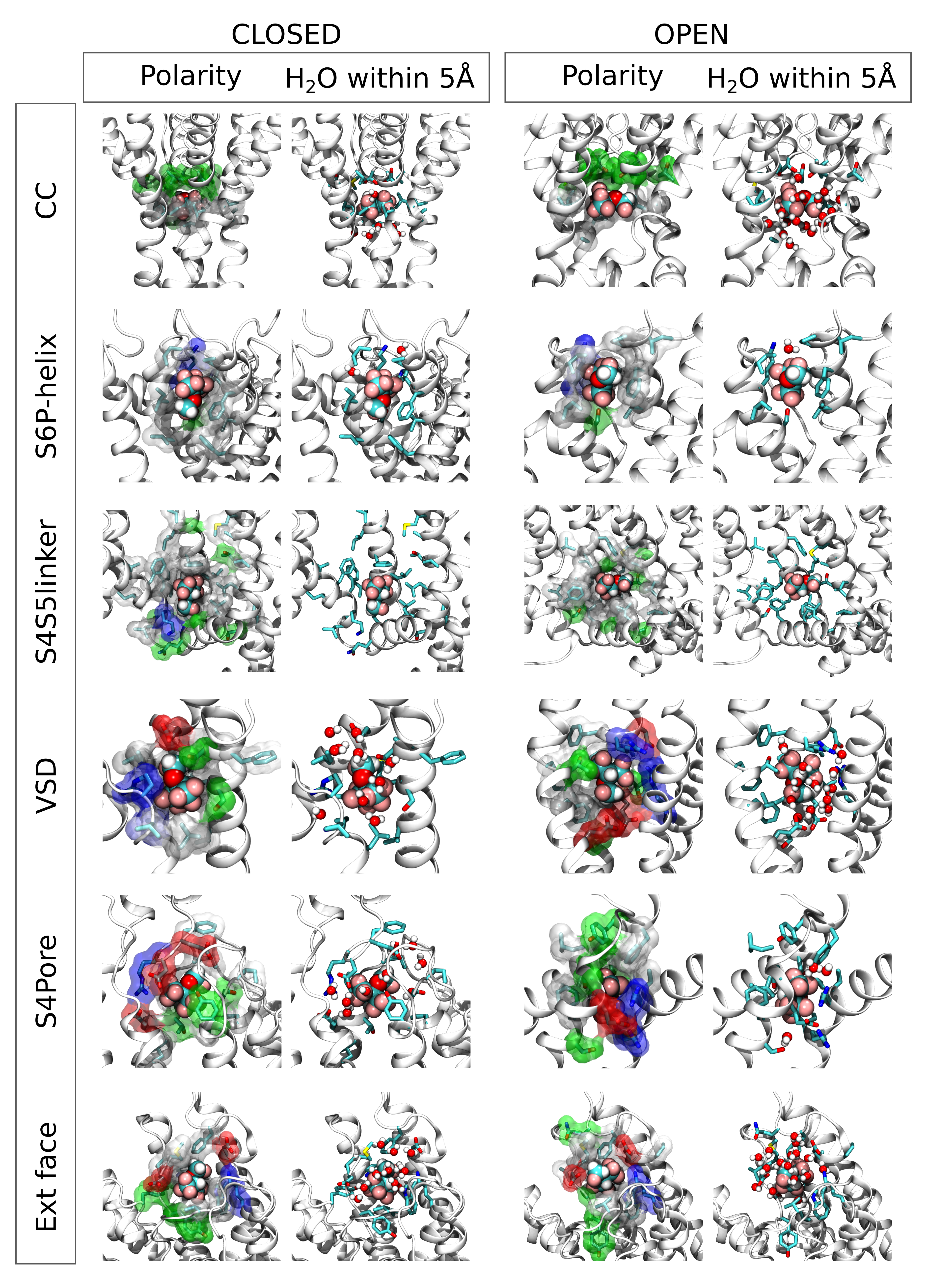

Supplement: S5 Fig — The first column of each conformation (C and O) displays a surface representation of the amino acids that compose the respective binding site, colored by their physical chemical character–white: apolar, green: polar non-charged, red: negatively charged and blue: positively charged. The second column shows the water molecules within a 5Å radius from the site’s geometrical center. Of note, sites S4S5 linker and S6P-helix are predominantly dehydrated and lipid accessible. (TIFF) [file pcbi.1006605.s005.tiff]

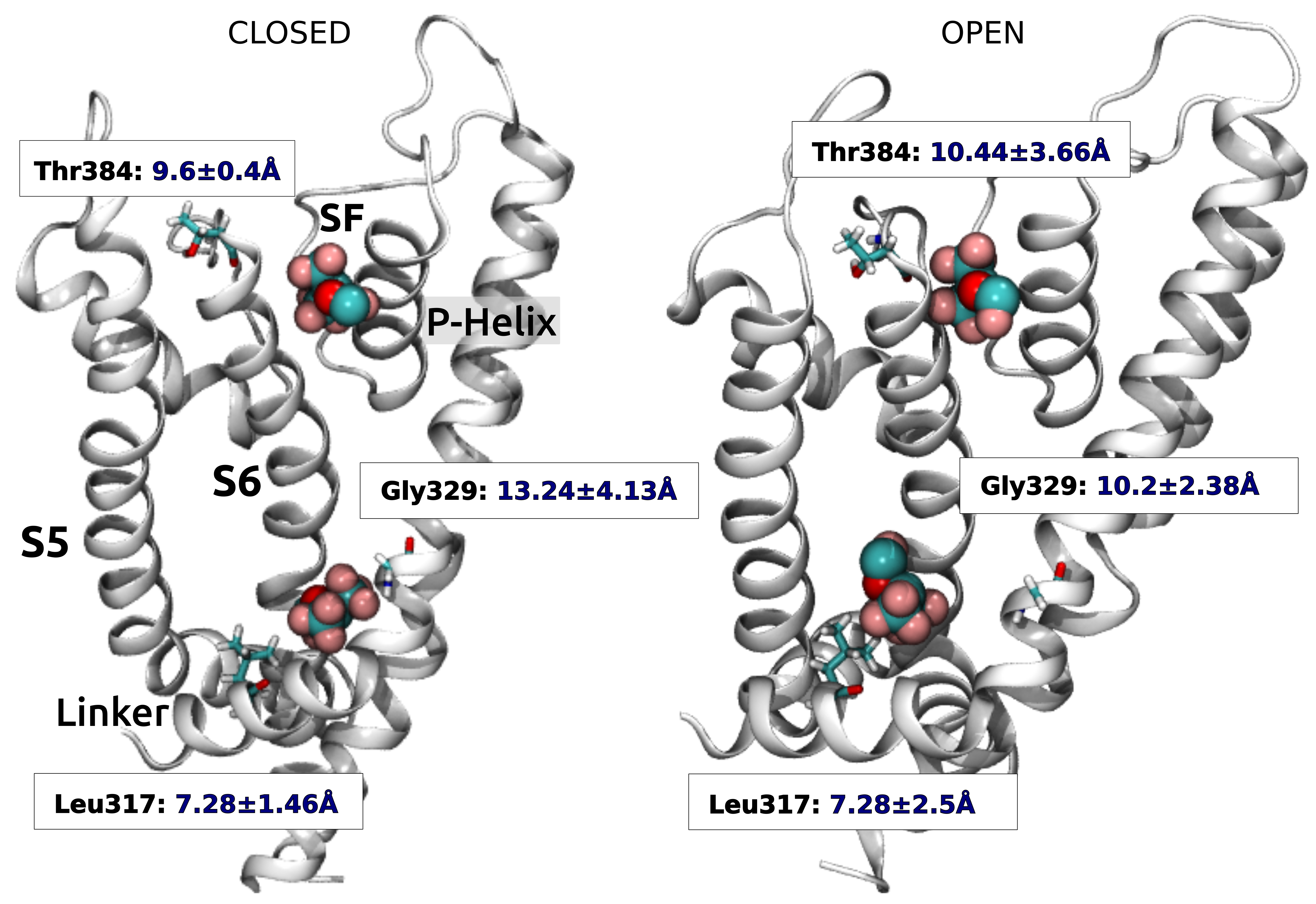

Supplement: S6 Fig — Shown are average distances between sevoflurane when bound to S6P-helix site and Thr384, and average distances of the ligand when bound to S4S5-linker site to residues Leu317 and G329. Distances were measured by considering both ensembles of equilibrium protein structures inputed into docking searches, and sevoflurane docking poses pertaining to a given binding site. (TIFF) [file pcbi.1006605.s006.tiff]

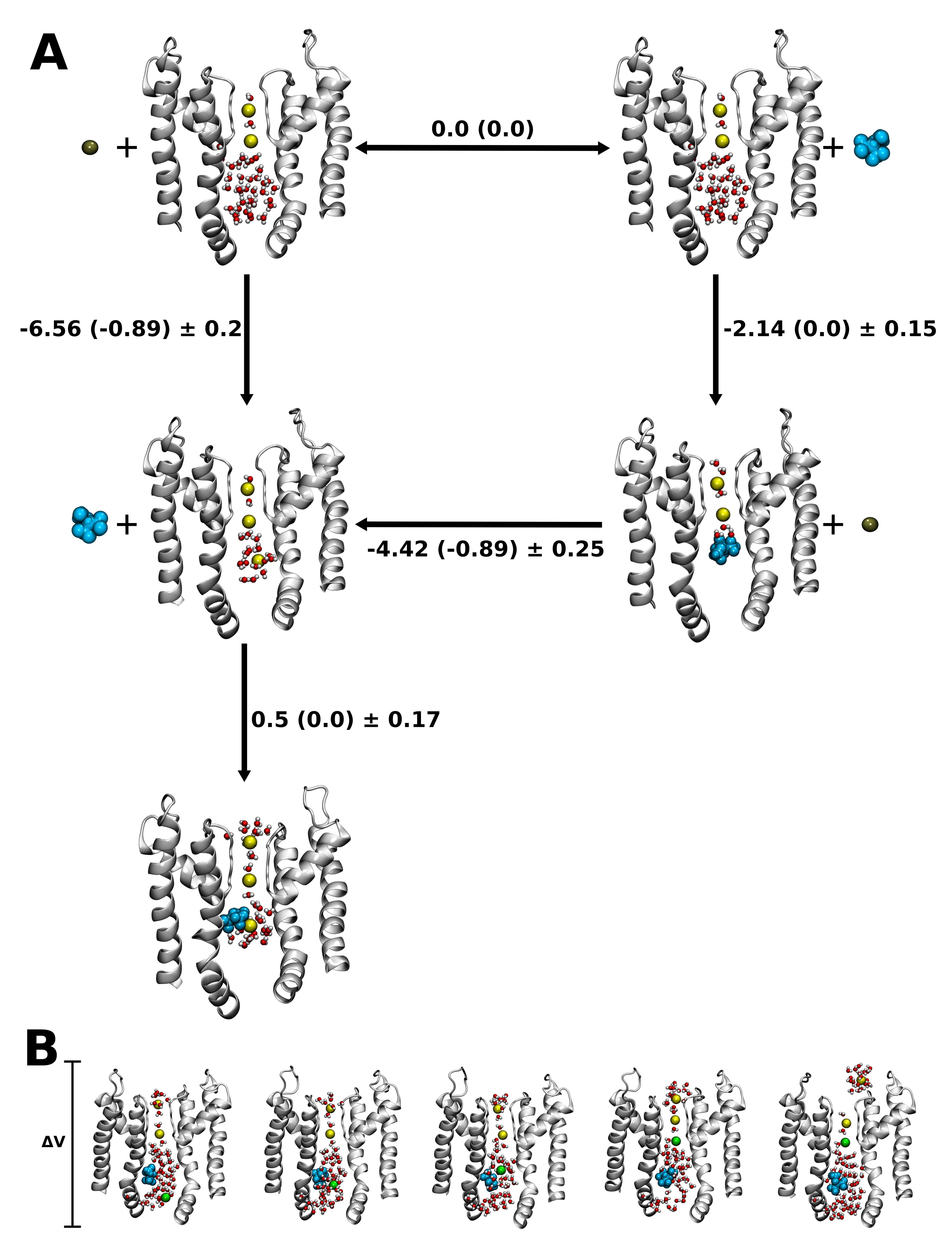

Supplement: S7 Fig — (A) Absolute and excess free-energies (kcal.mol-1) for binding potassium (yellow) and/or sevoflurane (blue) at the main pore of Kv1.2 (white). Excess free energies at 100mV are shown in parentheses. More favorable absolute and excess free-energies ensure single occupancy by potassium to be more likely than that by sevoflurane. In contrast, double occupancy by potassium and sevoflurane is unfavorable due to a positive absolute free energy for binding the molecule at the ion occupied cavity. (B) Shown are time-dependent trajectories of potassium ions diffusing through the open pore of Kv1.2 despite one bound sevoflurane molecule at the central cavity. The voltage-driven MD simulations were carried out at a depolarized potential of 600 mV to increase the rate of sampling of conduction events. Simulations spanned a total of ~ 30 ns. (TIFF) [file pcbi.1006605.s007.tiff]
