# Supplementary material for: Binding of the general anesthetic sevoflurane to ion channels
Source: PLoS Comput Biol. 2018 Nov 26;14(11):e1006605. doi: 10.1371/journal.pcbi.1006605 (PMC6283617; doi:10.1371/journal.pcbi.1006605)
Supplement: S1 Table — (PDF) [file pcbi.1006605.s011.pdf]

S1 Table. FEP calculations and equilibrium binding constants for singly- and doubly-occupied sites j of the closed channel structure.#

	Site	$k_C(1_j)$	$W^*_C(1_j) \pm \varepsilon$	$K_C(0_1...1_j...0_n)$	$\Delta G^\circ_C(0_1...1_j...0_n)$	$k_C(2_j)$	$W^*_C(2_j\|1_j) \pm \varepsilon$	$W^*_C(2_j) \pm \varepsilon$	$K_C(0_1...2_j...0_n)$	$\Delta G^\circ_C(0_1...2_j...0_n)$
**CC**	1	0.238	-5.4 ± 0.2	2.84E-01	-3.3	0.238	-5.0 ± 0.3	-10.4 ± 0.5	2.06E-02	-5.9
**S6P-helix**	2	0.069	-6.1 ± 0.3	5.93E+00	-5.2	0.080	-4.5 ± 0.2	-10.6 ± 0.5	1.01E+00	-8.2
	3	0.093	-5.3 ± 0.2	9.89E-01	-4.1	-	-	-	-	-
	4	0.171	-6.0 ± 0.2	1.29E+00	-4.2	0.144	-4.8 ± 0.2	-10.8 ± 0.4	1.49E-01	-7.1
	5	0.044	-4.4 ± 0.3	6.54E-01	-3.8	0.107	-4.4 ± 0.2	-8.8 ± 0.5	6.19E-02	-6.5
**S4S5linker**	6	0.052	-3.9 ± 0.3	2.25E-01	-3.2	0.063	-6.3 ± 0.2	-10.2 ± 0.5	1.00E+00	-8.2
	7	0.069	-6.1 ± 0.2	5.89E+00	-5.1	0.239	-7.1 ± 0.3	-13.2 ± 0.5	1.34E+01	-9.7
	8	0.244	-8.0 ± 0.2	2.20E+01	-5.9	0.103	-6.0 ± 0.2	-14 ± 0.4	2.97E+01	-10.2
	9	0.058	-6.4 ± 0.2	1.26E+01	-5.6	0.059	-6.7 ± 0.2	-13.1 ± 0.4	1.19E+02	-11.0
**VSD**	10	0.047	-1.6 ± 0.4	5.41E-03	-1.0	0.055	-3.7 ± 0.3	-5.3 ± 0.7	3.81E-04	-3.5
	11	0.049	-4.4 ± 0.2	5.57E-01	-3.7	0.147	-2.6 ± 0.3	-7.0 ± 0.5	1.34E-03	-4.3
	12	0.703	-5.5 ± 0.3	6.63E-02	-2.5	-	-	-	-	-
	13	0.034	-3.0 ± 0.3	9.35E-02	-2.7	0.025	-3.9 ± 0.3	-6.9 ± 0.6	3.44E-02	-6.2
**S4Pore**	14	0.106	-7.6 ± 0.2	3.92E+01	-6.3	0.098	-7.3 ± 0.2	-14.9 ± 0.4	5.03E+02	-11.9
	15	0.293	-5.4 ± 0.2	2.08E-01	-3.2	0.150	-5.4 ± 0.3	-10.8 ± 0.5	5.52E-02	-6.5
	16	0.047	-3.5 ± 0.4	1.30E-01	-2.9	-	-	-	-	-
	17	0.047	-0.8 ± 0.3	1.37E-03	-0.2	-	-	-	-	-
**Ext face**	18	1.356	3.2 ± 0.7	1.05E-08	6.8	-	-	-	-	-
	19	-	-	-	-	-	-	-	-	-
	20	-	-	-	-	-	-	-	-	-
	21	1.272	1.3 ± 0.5	2.85E-07	4.8	-	-	-	-	-

For singly occupied sites, units for $k_C(1_j)$, $W^*_C(1_j) \pm \epsilon$, $K_C(0...1_j...0)$ and $\Delta G^o_C(0...1_j...0)$ are kcal/mol/Å^2, kcal/mol, mM^{-1} and kcal/mol, respectively. For doubly occupied sites, units for $k_C(2_j)$, $W^*_C(2_j) \pm \epsilon$, $K_C(0...2_j...0)$ and $\Delta G^o_C(0...2_j...0)$ are kcal/mol/Å^2, kcal/mol, mM^{-2} and kcal/mol, respectively. FEP estimates, $W^*_C(1_j)$ and $W^*_C(2_j|1_j)$, and statistical errors ϵ were determined using the simple overlap sampling (SOS) formula (Lu et al., 2004) based on at least two independent FEP runs. $W^*_C(2_j)$ was computed as a two-step process $W^*_C(2_j) = W^*_C(1_j) + W^*_C(2_j|1_j)$ involving ligand coupling to a vacant site $W^*_C(1_j)$ followed by binding of a second ligand at the preoccupied site $W^*_C(2_j|1_j)$. Binding constants, $K_C(0...1_j...0)$ and $K_C(0...2_j...0)$, and the related binding free energies, $\Delta G^o_C(0...1_j...0)$ and $\Delta G^o_C(0...2_j...0)$, were quantified relative to a homogeneous and diluted aqueous solution occupied by ligands at an excess chemical potential of $\bar{\mu} = 0.10 \pm 0.09\, kcal.mol^{-1}$.
