# Supplementary material for: Binding of the general anesthetic sevoflurane to ion channels
Source: PLoS Comput Biol. 2018 Nov 26;14(11):e1006605. doi: 10.1371/journal.pcbi.1006605 (PMC6283617; doi:10.1371/journal.pcbi.1006605)
Supplement: S2 Table — (PDF) [file pcbi.1006605.s012.pdf]

S2 Table. FEP calculations and equilibrium binding constants for singly- and doubly-occupied sites j of the open channel structure.#

	Site	$k_O(1_j)$	$W^*_O(1_j) \pm \varepsilon$	$K_O(0_1 \ldots 1_j \ldots 0_n)$	$\Delta G^\circ_O(0_1 \ldots 1_j \ldots 0_n)$	$k_O(2_j)$	$W^*_O(2_j\|1_j) \pm \varepsilon$	$W^*_O(2_j) \pm \varepsilon$	$K_O(0_1 \ldots 2_j \ldots 0_n)$	$\Delta G^\circ_O(0_1 \ldots 2_j \ldots 0_n)$
CC	1	0.238	-4.1 ± 0.2	3.17E-02	-2.0	0.238	-5.6 ± 0.2	-9.7 ± 0.2	6.31E-03	-5.2
S6P-helix	2	0.243	-4.5 ± 0.2	6.02E-02	-2.4	0.155	-6.7 ± 0.2	-11.2 ± 0.4	1.46E-01	-7.1
	3	0.283	-4.7 ± 0.2	6.74E-02	-2.5	0.144	-4.8 ± 0.2	-9.5 ± 0.4	7.41E-03	-5.3
	4	0.253	-5.7 ± 0.2	4.29E-01	-3.6	0.142	-6.3 ± 0.2	-12 ± 0.4	6.01E-01	-7.9
	5	0.108	-3.8 ± 0.3	6.27E-02	-2.5	0.140	-5.1 ± 0.3	-8.9 ± 0.6	1.19E-02	-5.6
S4S5linker	6	0.045	-6.4 ± 0.2	1.84E+01	-5.8	0.105	-8.1 ± 0.2	-14.5 ± 0.4	8.49E+02	-12.2
	7	0.127	-7.8 ± 0.3	4.17E+01	-6.3	0.158	-7.3 ± 0.2	-15.1 ± 0.5	2.69E+02	-11.5
	8	0.050	-6.9 ± 0.2	3.70E+01	-6.2	0.109	-6.1 ± 0.2	-13 ± 0.4	5.55E+01	-10.6
	9	0.033	-6.7 ± 0.3	4.86E+01	-6.4	0.072	-5.2 ± 0.2	-11.9 ± 0.5	2.94E+01	-10.2
VSD	10	0.070	-3.5 ± 0.3	7.28E-02	-2.5	0.371	-2.7 ± 0.2	-6.2 ± 0.5	5.59E-05	-2.4
	11	0.112	-1.4 ± 0.4	1.04E-03	0.0	0.117	-1.3 ± 0.4	-2.7 ± 0.8	4.25E-07	0.5
	12	0.139	-3.3 ± 0.3	1.85E-02	-1.7	0.076	-4.7 ± 0.4	-8 ± 0.7	4.50E-03	-5.0
	13	0.111	-4.8 ± 0.3	3.25E-01	-3.4	0.098	-4.0 ± 0.4	-8.8 ± 0.7	1.65E-02	-5.8
S4Pore	14	0.137	-6.1 ± 0.2	2.11E+00	-4.5	0.074	-9.8 ± 0.3	-15.9 ± 0.5	2.92E+03	-12.9
	15	0.118	-2.2 ± 0.3	3.68E-03	-0.8	0.711	-0.5 ± 0.4	-2.7 ± 0.7	2.61E-08	2.2
	16	0.143	-7.9 ± 0.2	4.15E+01	-6.3	-	-	-	-	-
	17	0.113	-5.2 ± 0.2	6.22E-01	-3.8	0.303	-5.2 ± 0.3	-10.4 ± 0.5	4.39E-02	-6.3
Ext face	18	0.530	1.5 ± 0.4	7.57E-07	4.3	-	-	-	-	-
	19	0.453	-0.8 ± 0.4	4.63E-05	1.8	-	-	-	-	-
	20	0.283	-0.8 ± 0.4	9.36E-05	1.4	-	-	-	-	-
	21	0.332	-4.2 ± 0.5	2.28E-02	-1.9	-	-	-	-	-

For singly occupied sites, units for $k_O(1_j)$, $W^*_O(1_j) \pm \epsilon$, $K_O(0 \ldots 1_j \ldots 0)$ and $\Delta G^o_O(0 \ldots 1_j \ldots 0)$ are kcal/mol/Å², kcal/mol, mM^{-1} and kcal/mol, respectively. For doubly occupied sites, units for $k_O(2_j)$, $W^*_O(2_j) \pm \epsilon$, $K_O(0 \ldots 2_j \ldots 0)$ and $\Delta G^o_O(0 \ldots 2_j \ldots 0)$ are kcal/mol/Å², kcal/mol, mM^{-2} and kcal/mol, respectively. FEP estimates, $W^*_O(1_j)$ and $W^*_O(2_j|1_j)$, and statistical errors ϵ were determined using the simple overlap sampling (SOS) formula (Lu et al., 2004) based on at least two independent FEP runs. $W^*_O(2_j)$ was computed as a two-step process $W^*_O(2_j) = W^*_O(1_j) + W^*_O(2_j|1_j)$ involving ligand coupling to a vacant site $W^*_O(1_j)$ followed by binding of a second ligand at the preoccupied site $W^*_O(2_j|1_j)$. Binding constants, $K_O(0 \ldots 1_j \ldots 0)$ and $K_O(0 \ldots 2_j \ldots 0)$, and the related binding free energies, $\Delta G^o_O(0 \ldots 1_j \ldots 0)$ and $\Delta G^o_O(0 \ldots 2_j \ldots 0)$, were quantified relative to a homogeneous and diluted aqueous solution occupied by ligands at an excess chemical potential of $\bar{\mu} = 0.10 \pm 0.09\, kcal.mol^{-1}$.
